# Siponimod treatment response shows partial BDNF dependency in multiple sclerosis models

**DOI:** 10.1038/s41598-024-68715-x

**Published:** 2024-08-01

**Authors:** Hasan Hüseyin Hendek, Alina Blusch, Neele Heitmann, Sarah Oberhagemann, Seray Demir, Xiomara Pedreiturria, Ralf Gold, Simon Faissner

**Affiliations:** Department of Neurology, Ruhr-University Bochum, St. Josef-Hospital, Gudrunstr. 56, 44791 Bochum, Germany

**Keywords:** Multiple sclerosis, Siponimod, Brain derived neurotrophic factor, Experimental autoimmune encephalomyelitis, Neuroprotection, Neuroinflammation, Diseases of the nervous system, Multiple sclerosis, Multiple sclerosis

## Abstract

So far, only a small number of medications are effective in progressive multiple sclerosis (MS). The sphingosine-1-phosphate-receptor (S1PR)-1,5 modulator siponimod, licensed for progressive MS, is acting both on peripheral immune cells and in the central nervous system (CNS). So far it remains elusive, whether those effects are related to the neurotrophin brain derived neurotrophic factor (BDNF). We hypothesized that BDNF in immune cells might be a prerequisite to reduce disease activity in experimental autoimmune encephalomyelitis (EAE) and prevent neurotoxicity. MOG_35–55_ immunized wild type (WT) and BDNF knock-out (BDNF^ko^) mice were treated with siponimod or vehicle and scored daily in a blinded manner. Immune cell phenotyping was performed via flow cytometry. Immune cell infiltration and demyelination of spinal cord were assessed using immunohistochemistry. In vitro*,* effects on neurotoxicity and mRNA regulation were investigated using dorsal root ganglion cells incubated with EAE splenocyte supernatant. Siponimod led to a dose-dependent reduction of EAE scores in chronic WT EAE. Using a suboptimal dosage of 0.45 µg/day, siponimod reduced clinical signs of EAE independent of BDNF-expression in immune cells in accordance with reduced infiltration and demyelination. Th and Tc cells in secondary lymphoid organs were dose-dependently reduced, paralleled with an increase of regulatory T cells. In vitro, neuronal viability trended towards a deterioration after incubation with EAE supernatant; siponimod showed a slight rescue effect following treatment of WT splenocytes. Neuronal gene expression for CCL2 and CX3CL1 was elevated after incubation with EAE supernatant, which was reversed after siponimod treatment for WT, but not for BNDF^ko^. Apoptosis markers and alternative death pathways were not affected. Siponimod exerts both anti-inflammatory and neuroprotective effects, partially related to BDNF-expression. This might in part explain effectiveness during progression in MS and could be a target for therapy.

## Introduction

Whereas there has been great success over the last decades regarding the development of medications for relapsing remitting multiple sclerosis (RRMS), the progressive forms of multiple sclerosis (MS) remain difficult to treat^1^. A limiting factor towards treatment of progressive forms of MS is insufficient understanding of the complex pathophysiology underlying progression. Currently, it seems most plausible to assume that compartmentalized chronic inflammation and neurodegeneration, occurring behind a closed blood–brain barrier (BBB), is mostly driven by activated microglia and, to a lesser extent, T cells, iron accumulation and oxidative stress^2^. Additionally, studies in murine MS models such as experimental autoimmune encephalomyelitis (EAE), show an imbalance of glutamatergic activity and GABAergic inhibition resulting in an inflammation-based synaptopathy. This results in neurodegeneration in grey matter (GM) independent of demyelination^3^. While disease modifying therapy (DMT) treatment in RRMS is more effective, those approaches have been less successful at reducing progression in progressive forms of MS, as shown e.g. with the negative trial using the sphingosine-1-phosphate receptor (S1PR) modulator fingolimod^4^. The S1PR-1,5 modulator siponimod, a second generation S1P agonist^5^, has shown significant effects in reducing the risk of 3-month confirmed disability progression and brain atrophy in secondary progressive multiple sclerosis (SPMS) patients compared to placebo in the EXPAND study^6^, and has been the first medication to be approved by the FDA as treatment for SPMS in 2019. Currently, it is approved for RRMS, clinically-isolated syndrome and active SPMS in the US, active SPMS in Europe and SPMS in Australia and Japan^7,8^.

While several mechanisms are understood, especially effects in the CNS are known to lesser extent. The lysophospholipid sphingosine-1-phosphate (S1P) acts on G-protein-coupled receptors that are characterised as S1PR-1 to S1PR-5. These subtypes can be found on a multitude of tissues and present multifaceted effects on immune cells, cardiovascular function, and in the central nervous system (CNS)^9,10^. S1P pathways have been linked to various diseases including cancer and MS with elevated S1P levels in cerebrospinal fluid and brain parenchyma^7^. The S1PR-1, targeted by siponimod, has a paramount importance in neuromodulation and immunomodulation, as it can be found on neurons, astrocytes, and oligodendrocytes as well as on immune cells like microglia and lymphocytes^7^. Siponimod serves as a functional antagonist, as S1PR-1 is internalised and degraded after binding^5,11^. Thus, it is assumed that siponimod both ameliorates neuroinflammation as well as neurodegeneration^12^. Neuroinflammation is targeted by preventing lymphocyte egress from lymph nodes, leading to a reduction of lymphocyte circulation. Due to its short half-life of 30 h, a return to normal peripheral lymphocyte levels is observed within one week^3,5^. Additionally, siponimod stops chemoattraction and reduces lymphocytic infiltration into the CNS by stabilising the BBB in inflammatory areas.

The effects of siponimod on neurodegeneration are still subject of study. MS and EAE lesions are marked by a loss of parvalbumine-positive interneurons, resulting in a striatal synaptopathy due to a reduction of GABAergic inhibition and glutamatergic overexcitation, leading to neurodegeneration. Siponimod crosses the BBB and has potentially direct effects on neurons and glia. Thus, it preserves parvalbumine-positive interneurons and balances glutamatergic excitation^3^. Furthermore, S1PR modulators decrease ferritin levels and NO metabolites as well as reduce key cytokines like IL-6 and RANTES in microglia, which corresponds to reduced proinflammatory microglial activation^3,13^. Additionally, axonal remyelination is induced by S1PR-1 expressing oligodendrocyte progenitor cells in damaged brain tissue, reconstituting physiological conditions^14^.

We set out to investigate the importance of *brain derived neurotrophic factor* (BDNF) on the treatment response to siponimod. Neurotrophins like BDNF modulate neuronal growth and neuronal and axonal protection. Low BDNF levels correlate with resurgence of neuropathological diseases. Attempts to directly insert BDNF have failed due to its short half-life. However, fingolimod, and more recently, siponimod lead to higher BDNF levels in striatal and cortical brain tissue in mice^15–17^. More specifically, fingolimod leads to increased BDNF production in neurons, astrocytes, and microglia^18,19^. Additionally, S1PR activation necessitates modulation of BDNF and neurotrophins via extracellular signal-regulated kinase (ERK)1/2/mitogen activated protein kinase (MAPK) and akt phosphorylation, downregulating neuronal apoptosis and cell death^20^. BDNF also shows positive modulation of neuronal dendritic structure due to cAMP response-element binding protein, resulting in an increasing number of c-Fos positive neurons.

In this study, we set out to investigate the functional role of BDNF for the therapeutic effect of siponimod to better understand siponimod´s mechanism of action with potential for further development as a therapeutic target in MS.

## Methods

### Experimental autoimmune encephalomyelitis

All animal experiments were approved by the animal care and ethics committee in Düsseldorf Nordrhein-Westfalen; Germany (LANUV, no. 82-02.04.2019-A425). All methods were carried out in accordance with official guidelines and regulations and comply with ARRIVE guidelines. Mice were kept under environmentally controlled conditions at the animal facility of the Medical Faculty, Ruhr-University Bochum^21^. C57BL6/J mice (Charles River, Wilmington, MA, USA) and partially BDNF-deficient mice on the same genetic background between 8 and 12 weeks were used for the experiments. Weight, age, and sex between groups were matched for each experiment. Partial BDNF-deficiency in mice (BDNF^ko^ mice) was created with a Cre-loxP-model. Mice with a floxed BDNF^fl/fl^ gene in the coding exon 8^22^ were crossed with mice expressing Cre-recombinase controlled by lysM-Cre promoter specific for myeloid cells^23,24^ and the CD4-Cre promoter specific for T-Helper cells^25^. Thus, BDNF^ko^ mice have reduced BDNF expression in macrophages/microglia and T-Helper cells, both for infiltrating leukocytes as well as resident cells in lymphoid organs (Fig. S1)^23,26^. Phenotypically, BDNF^ko^ mice show no clinical or behavioural impairment.

Mice were injected with 50–100 µg Myelin Oligodendrocyte Glycoprotein_35–55_ (MOG_35–55_, Genosphere Biotechnologies, Paris, France) mixed with 200 µg complete Freund`s Adjuvant (CFA) subcutaneously into both hind flanks to induce experimental autoimmune encephalomyelitis (EAE)^21,23^. 250 ng pertussis toxin (PTX, Sigma Aldrich GmbH, St. Louis, MO, USA) dissolved in PBS were injected intraperitoneally on days 0 and 2. Mice were treated with siponimod (MedChemExpress, South Brunswick Township, NJ, USA) in rape seed oil or vehicle daily via oral gavage in a blinded manner. Mice were weighted and scored daily using a 10-point-scale^21^. Mice with a score of 7 or a weight loss of > 20% were euthanized using cervical dislocation^27^. At the end of the experiment, mice were euthanised with carbondioxide inhalation according to AVMA guidelines^27,28^. Their blood, spleen, lymph nodes and spinal cord were extracted. Blood, spleen, and lymph node cells were prepared for flow cytometry. Histology and immunohistochemistry were performed with spinal cord slices and spleen.

### Flow cytometry

Blood, spleen and lymph nodes were processed as described^21^. Leukocytes were stained with antibodies conjugated with fluorochromes for flow cytometry (Table S1) of 100,000 events using BD FACSCelesta™ (BD, Heidelberg, Germany). Data were obtained using BDFACSDiva software (BD, Heidelberg, Germany) and analysed via FlowJo (Becton Dickinson & Company 2006–2021, Ashland, USA).

### Histochemistry for leukocyte infiltration and demyelination

Spinal cord was divided into cervical, thoracic, and lumbar parts. Spinal cord and spleen were fixated and embedded into paraffin. Slices were deparaffinized with Roti-Histol (Carl-Roth GmbH+ Co. KG, Karlsruhe, Germany) and dehydrated in a descending ethanol chain.

Hematoxylin (SigmaLifeScience, Sigma Aldrich, Co., St. Louis, USA) and eosin (Carl-Roth GmbH+ Co. KG, Karlsruhe, Germany) staining was performed to assess leukocyte infiltration, and Luxol’s^®^ Fast Blue (LFB; ACROS Organics, New Jersey, USA) and Schiff`s Base (Carl Roth GmbH+ Co. KG, Karlsruhe, Germany) staining to assess demyelination.

For BDNF-knockout in macrophages/monocytes, we stained paraffin slices of spleen and spinal cord with chicken anti-Iba1 (SynaticSystems GmbH, Goettingen, Germany) and with secondary antibody CY3 (MerckMillipore, Darmstadt, Germany). To show BDNF-knockout in T cells, we stained paraffin slices with rat anti-mouse CD4 (Thermo Fisher Scientific, Eugene, OR, USA) and secondary antibody Alexa Fluor® 488 (Thermo Fisher Scientific, Eugene, OR, USA). We stained BDNF using rabbit anti-BDNF (MerckMillipore, Darmstadt, Germany) and secondary antibody Cy5 (MerckMillipore, Darmstadt, Germany) (Table S2).

Pictures of slices were acquired with a Pecon® microscope (Carl Zeiss Microscopy GmbH, Oberkochen, Germany) using ZEN 3.3 (blue edition, version 3.3.89.0000, Carl Zeiss Microscopy GmbH, Oberkochen, Germany). For infiltration, the percentage of white matter area that was infiltrated by leukocytes, shown by their stained cell nuclei, was measured. For demyelination, the percentage of white matter stained by Schiff`s base instead of LFB was measured. To assess BDNF-knockout, the percentage of Iba1^+^ BDNF^+^ or CD4^+^ BDNF^+^ double positive cells in relation to Iba1^+^ or CD4^+^ single positive cells was manually counted and calculated. The percentages were then normalized to WT value. The images were blinded and analysed with ImageJ (version 1.535, Wayne Rasband and Co., national institute of health, USA).

### Dorsal root ganglion cell culture

Dorsal root ganglions (DRG) were obtained from mouse E12.5 embryos as previously described^29^. The DRG were digested with type I collagenase (Sigma Aldrich GmbH, Steinheim, Germany) for 45 min at 37 °C and 5% CO_2_. Using OptiPrep gradient (Sigma Aldrich GmbH, Steinheim, Germany) DRG neurons were isolated and 1.5 × 10^4^ cells per well were plated on Poly-D-lysine (PDL, Sigma Aldrich GmbH, Steinheim) and laminin (Sigma Aldrich GmbH, Steinheim, Germany) coated 96-well-plates in DRG medium (Table S3) at 37 °C and 5% CO_2_.

Spleens from EAE mice 10 days post injection were explanted and sieved through 40 µm strainers. The splenocytes were separated via density gradient centrifugation with Ficoll® (Cytiva, Marlborough, MA, USA) and 2 × 10^5^ lymphocytes were cultured on each 96-well plate in 200 µl splenocyte medium (Table S4). After 24 h, conditioned splenocyte supernatant was applied on DRG neurons in 96-well plates. After incubation of 24 h, viability and cell death analysis was performed for DRG neurons and splenocytes. Additionally, qPCR analysis and β-III-tubulin immunocytochemical staining was performed for DRG neurons.

### Viability and cell death analysis of DRG neurons and splenocytes

For viability, CellTiter-Glo® 2.0 assay, measuring ATP quantity, (Promega, Madison, WI, USA) and Caspase-Glo® 3/7 assay (Promega, Madison, WI, USA) were used according to manufacturer instructions. The luminescence measurement was performed using CLARIOstar® Plus (BMG Labtech, Ortenberg, Germany).

### qPCR analysis of DRG neurons

Neuronal RNA was extracted using RNeasy™ Plus Mini Kit (Qiagen, Venlo, Netherlands) and cDNA was transcribed using GoScript™ Reverse Transcriptase synthesis kit (Promega, Madison, WI, USA). For qPCR experiments GoTaq® qPCR (Promega, Madison, WI, USA) and QuantStudio3 qPCR cycler (applied biosystems by ThermoFisher Scientific, Waltham, MA, USA) were utilized. For each primer, experiments were performed in duplicates and normalized to the housekeeping genes β-actin and 18-S (Table S5). ΔΔCT values were calculated and QuantStudioTM Design& Analysis software v1.3.1 (ThermoFisher Scientific, Waltham, MA, USA) was used for data analysis.

### β-III-tubulin staining of DRG neurons

DRG neurons were fixed with 4% PFA, β-III-tubulin (T-2200, (Sigma Aldrich GmbH, Steinheim, Germany) and secondary antibody Alexa Fluor® 488 (ThermoFisher Scientific, Waltham, MA, USA) were used for immunocytochemical staining and cells were mounted with DAPI fluoromount-G(R) (Biozol, Eching, Germany) (Table S5).

### Statistics

Data were analysed using Prism software V.9 (GraphPad Software, San Diego, CA, USA). Non-parametric data were analysed using Kruskal–Wallis test with post-hoc analysis using Dunn`s multiple comparisons test as shown in respective figure legends. Correlations were analysed using non-parametric Spearman correlation. Data are presented as mean values with standard error of the mean (SEM). A p-value < 0.05 was considered as statistically significant.

### Ethics approval

All animal experiments were approved by the animal care committee in Düsseldorf Nordrhein-Westfalen; Germany (LANUV, no. 82-02.04.2019-A425).

## Results

### Siponimod improves clinical signs of experimental autoimmune encephalomyelitis

**Figure 1:**
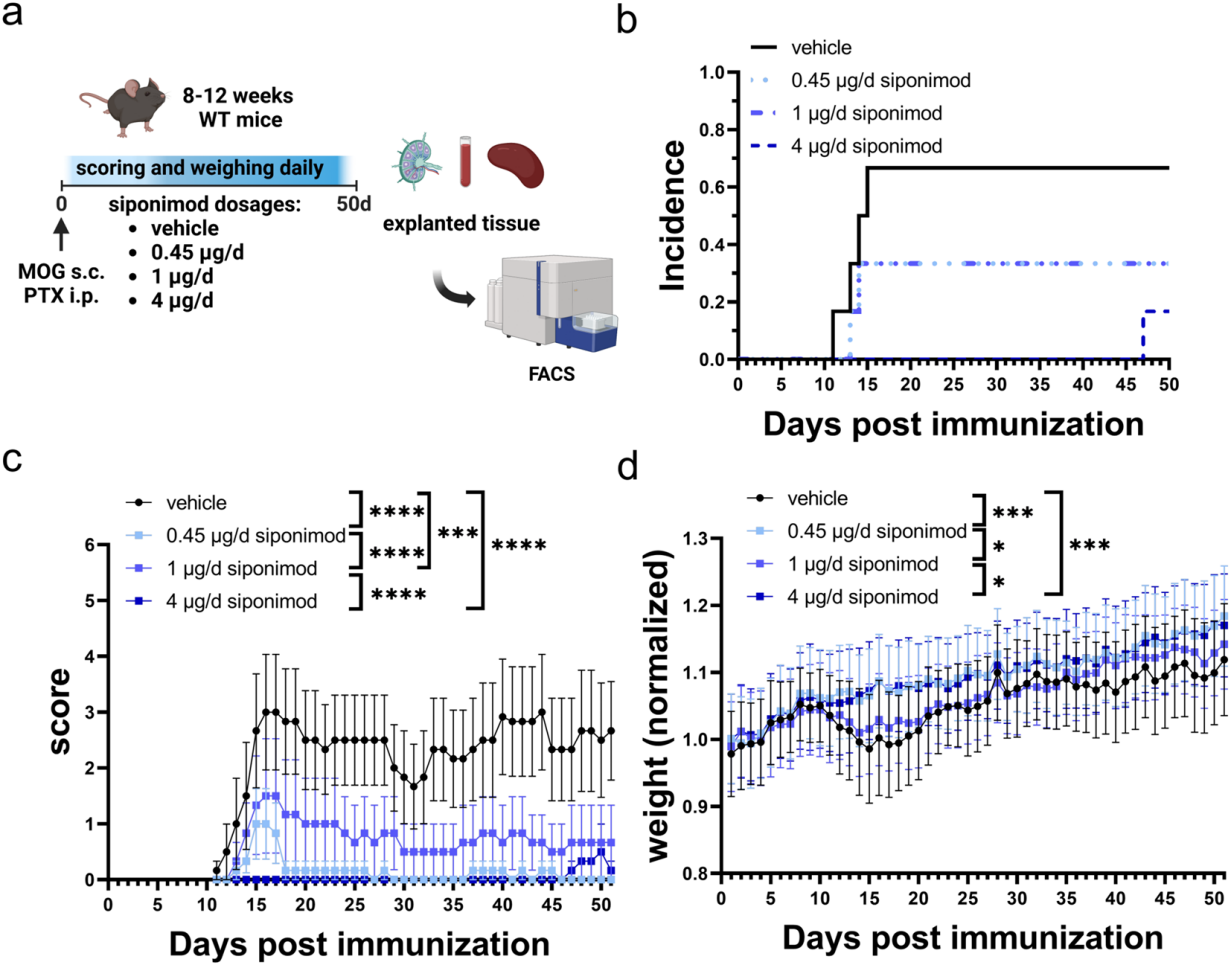
Prophylactic siponimod dose-dependently ameliorates chronic MOG-EAE in C57BL6 mice. (**a**) 8–12 week old C57BL6 mice were immunized with MOG_35-55_ and treated with PTX on days 0 and 2. Mice were treated with vehicle or different dosages of siponimod by oral gavage from day 0 to 50. Created with BioRender.com. (**b**) Incidence and (**c**) clinical scores showed a significant reduction for siponimod treatment in all groups compared to vehicle. (**d**) Weight loss was significantly lower in 0.45 µg/day and 4 µg/day siponimod treated mice compared to the vehicle group. n = 6 for each group. Data are shown as mean ± SEM. Weight was normalized to respective group mean. Incidence data (**b**) were analyzed with Logrank test. Score and weight data (**c**, **d**) were analyzed for normality with Shapiro–Wilk test and for significance with Kruskal–Wallis test with post-hoc analysis using Dunn’s multiple comparisons test. Significances are depicted as *p < 0.05; ***p < 0.001; ****p < 0.0001. *WT* wildtype, *MOG* Myelin oligodendrocyte glycoprotein, *s.c.* subcutaneously, *PTX* pertussis toxin, *i.p.* intraperitoneally.

Across different studies, different applications and dosing regimens of siponimod in EAE have been conducted^3,30,31^. To assess the optimal dosage for treatment of BDNF^ko^ mice, we treated C57BL6 wildtype (WT) mice prophylactically with three different dosages of 0.45 µg/day, 1 µg/day and 4 µg/day siponimod (Fig. 1a). As expected, siponimod lowered incidence by 66.7% in vehicle mice compared to 16.7% in the 4 µg group. This was reflected in a delay of clinical onset by 2 days in the two lower siponimod groups compared to the control group. In the 4 µg group, clinical signs only developed after day 47 (Fig. 1b). Siponimod also reduced overall disability as shown with clinical scores. At the end of the experiment, vehicle treated mice had a score of 2.5 ± 0.9 (mean ± SEM) compared to 0—0.66 in the treated groups (± 0–0.45) (p < 0.0001; Fig. 1c). Additionally, siponimod treated mice suffered less weight loss as a sign of general well-being (Fig. 1d). The dosage of 1 µg/day proved to be less effective at reducing EAE signs compared to the 0.45 µg group (p < 0.0001), which we attribute to one mouse from 1 mg/day group not benefitting from siponimod. Omitting the outlier showed similar beneficial effects of both dosages (Fig. S2).

### Siponimod affects lymphocyte populations in blood, spleen and lymph nodes

**Figure 2:**
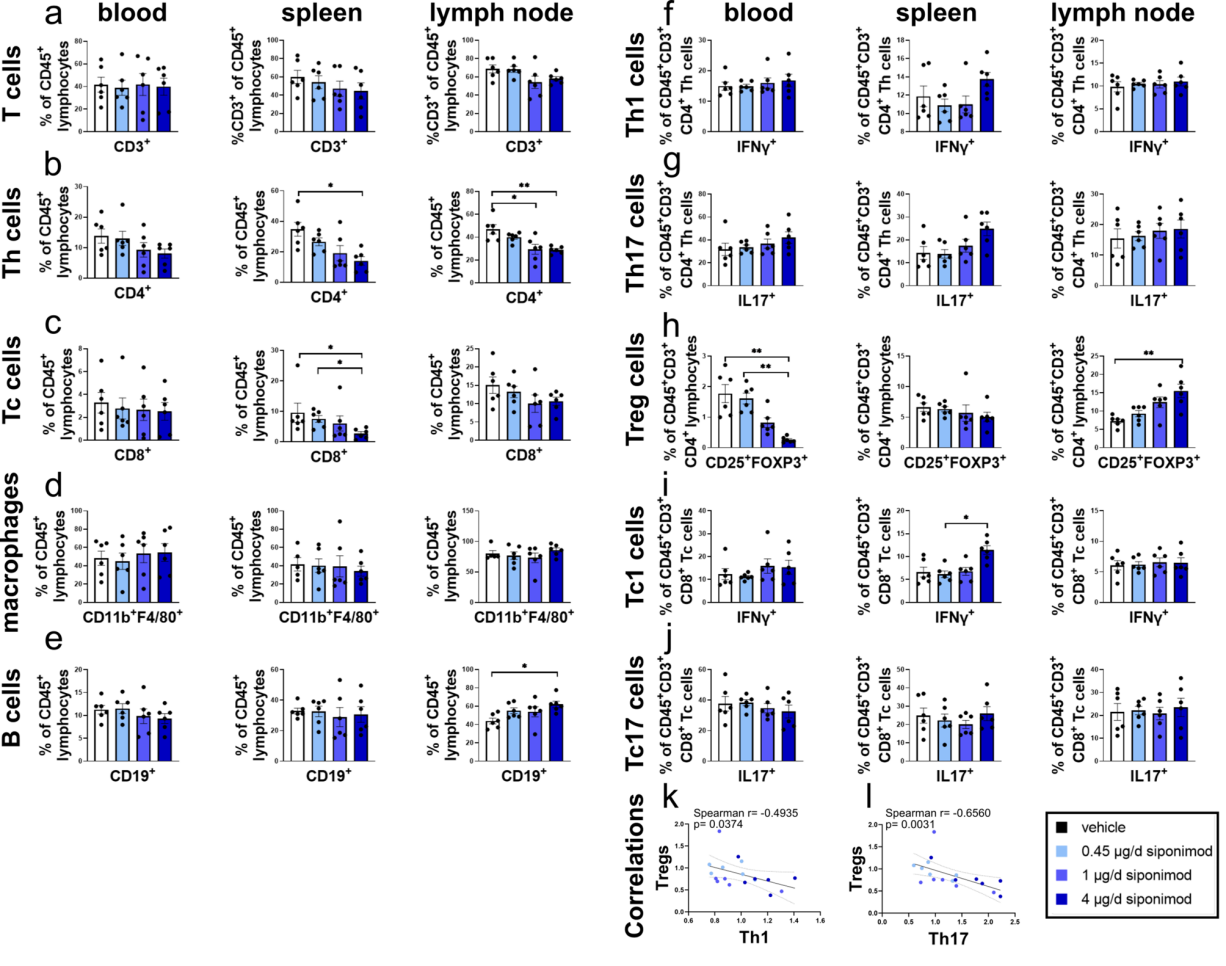
Siponimod dose-dependently modifies the T cell compartment towards a regulatory state. Cells from blood, spleen and lymph nodes of siponimod or vehicle-treated EAE mice were analyzed via flow cytometry. T cell subpopulations were related to parent. (**a**) Whereas T cells showed no regulation due to siponimod treatment, there was (**b**) a reduction of frequencies in spleen and lymph nodes for Th cells and (**c**) in spleen for Tc cells. (**d**) Macrophage populations were not affected. (**e**) B cells in blood and spleen were unaffected, while showing an increase in lymph nodes. (**f**, **g**) Th1 and Th17 populations did not show significant changes. (**h**) Siponmod led to a dose-dependent increase in Treg cell frequenecy in blood, and a decrease in lymph nodes. (**i**) Tc1 cell frequency was elevated in spleen, and (**j**) Tc17 frequency showed no change for siponimod treatment. (**k**, **l**) Th1 and Th17 cells were restrained dose-dependently in spleen, showing an inverse correlation with Treg frequencies. n = 6 for each group. Data are shown as mean ± SEM. Flow cytometry data (**a**–**j**) were analyzed for normality with Shapiro–Wilk test and for significance with Kruskal–Wallis test with post-hoc analysis using Dunn`s multiple comparisons test. Correlation analysis (**k**, **l**) was performed using Spearman correlation and shown with 95% confidence bands. Significances are depicted as *p < 0.05; **p < 0.01. *Th cells* T helper cells, *Tc cells* cytotoxic T cells, *Treg cells/Tregs* T regulatory cells.

Siponimod is well known to reduce lymphocyte circulation due to modulation of chemotaxis and hindering lymphocyte egress from lymph nodes^12^. Here, we investigated effects of siponimod on different immune sites including blood, spleen, and lymph nodes at the end of the experiment. We saw no effect on T cell frequencies (Fig. 2a). Regarding T-Helper cells (Th cells) and cytotoxic T cells (Tc cells), siponimod led to a decrease both in spleen (p = 0.0172 for Th cells, p = 0.0478 for Tc cells; Fig. 2b,c), and, for Th cells, in lymph nodes (p = 0.0093 for 4 µg/d vs. control, p = 0.0422 for 1 µg/day vs. control). Siponimod had no effect on frequencies of macrophages in blood, spleen and lymph nodes (Fig. 2d). B cell frequencies were increased in lymph nodes in the highest siponimod group compared to control while frequencies in blood and spleen were unaltered (p < 0.05; Fig. 2e).

Subpopulations of Th and Tc cells are commonly differentiated into autoreactive IFN-y- positive (Th1 and Tc1 cells) and IL-17- positive T cells (Th17 and Tc17 cells) as well as protective regulatory T cells (Tregs). Siponimod did not induce a significant regulation regarding the frequencies of Th1 and Th17 populations (Fig. 2f,g). Tregs showed a dose-dependent reduction of frequencies in blood (p = 0.0017 for 4 µg/day vs. control; p = 0.0031 for 4 µg/day vs. 0.45 µg/day; Fig. 2h), and an increase in lymph nodes (p = 0.0087 for 4 µg/day vs. control). Surprisingly, siponimod did not induce any significant lymphocyte regulation in blood, except for Tregs. Tc1 and Tc17 cell populations were not altered (Fig. 2i,j), except for an increase of Tc1 cells in spleen (p = 0.0256 for 4 µg/day vs. 0.45 µg/day; Fig. 2i).

Strikingly, increasing frequencies of Th1 and Th17 both correlated with reduced frequencies of Tregs in spleen (Th1: r = − 0.4935, p = 0.0374; Th17: r = − 0.6560, p = 0.0031; Fig. 2k,l), indicating an affinity for siponimod to retain autoreactive cells more effectively in lymphatic organs than autoimmunoprotective Tregs.

### Siponimod improves clinical signs independent from BDNF in immune cells

**Figure 3:**
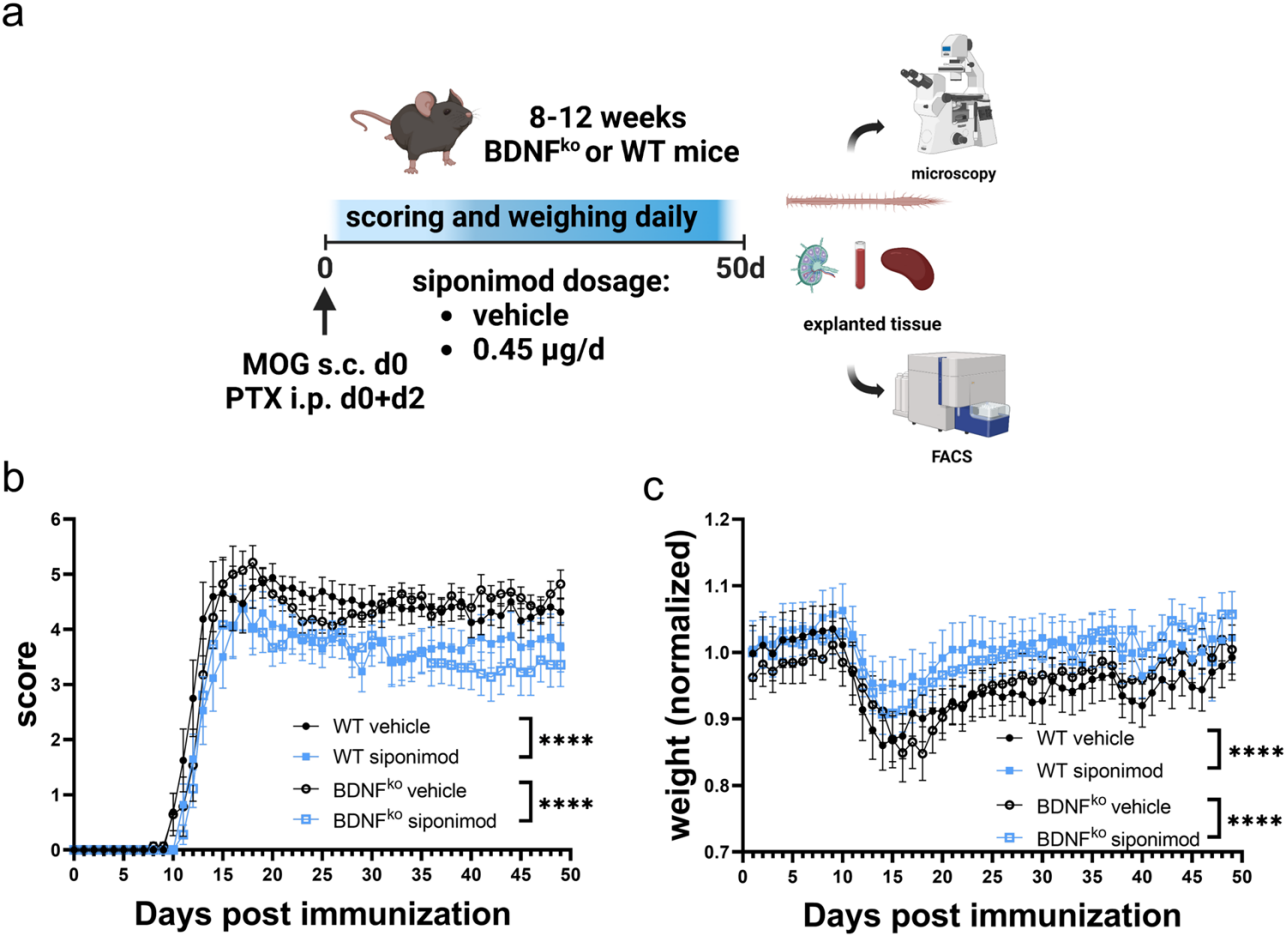
Treatment with siponimod ameliorates MOG-EAE in control and BDNF^ko^ mice. (**a**) MOG-immunized WT and BDNF^ko^ mice (8–12 weeks) were administered with 0.45 µg/d siponimod or vehicle daily for 50 days. Created with BioRender.com. (**b**) Clinical scores were ameliorated and (**c**) weight loss was averted in siponimod treated mice independent of BDNF^ko^. Shown are pooled data of n = 3 independent experiments. WT vehicle: n = 15, WT siponimod: n = 17, BDNF^ko^ vehicle: n = 15, BDNF^ko^ siponimod: n = 18. Scores of four mice without EAE-symptoms (WT vehicle: n = 2, WT siponimod: n = 1, BDNF^ko^ vehicle: n = 1) and three mice with premature exitus (BDNF^ko^ vehicle: n = 3). were excluded. Data are shown as mean ± SEM. Weight was normalized to group mean. Data were analyzed for normality with Shapiro–Wilk test and tested for significance with Kruskal–Wallis test with post-hoc analysis using Dunn’s multiple comparisons test. Significances are depicted as ****p < 0.0001. BDNF^ko^ = partial BDNF knock-out.

For the investigation of siponimod in BDNF^ko^ mice we used the suboptimal dosage of 0.45 µg/day, since this dosage elicited clear, but suboptimal clinical effects without suppressing EAE completely (Fig. 3a). Prophylactic treatment of BDNF^ko^ mice following induction of EAE improved clinical signs significantly both for WT and BDNF^ko^ mice compared to their respective controls (p < 0.0001 for WT and BDNF^ko^; Fig. 3b). Interestingly, BDNF-deficiency had no effect on clinical scores (Fig. 3b). These results were corroborated by similar effects on weight loss. Control mice lost 9–13% of their starting weight at peak of disease, while siponimod treated mice lost significantly less weight (3–5%; p < 0.0001 for WT and BDNF^ko^; Fig. 3c). Thus, BDNF-deficiency did not mitigate the positive clinical effect of siponimod in EAE.

### Siponimod reduces immune cell infiltration and demyelination independent of BDNF in immune cells

**Figure 4:**
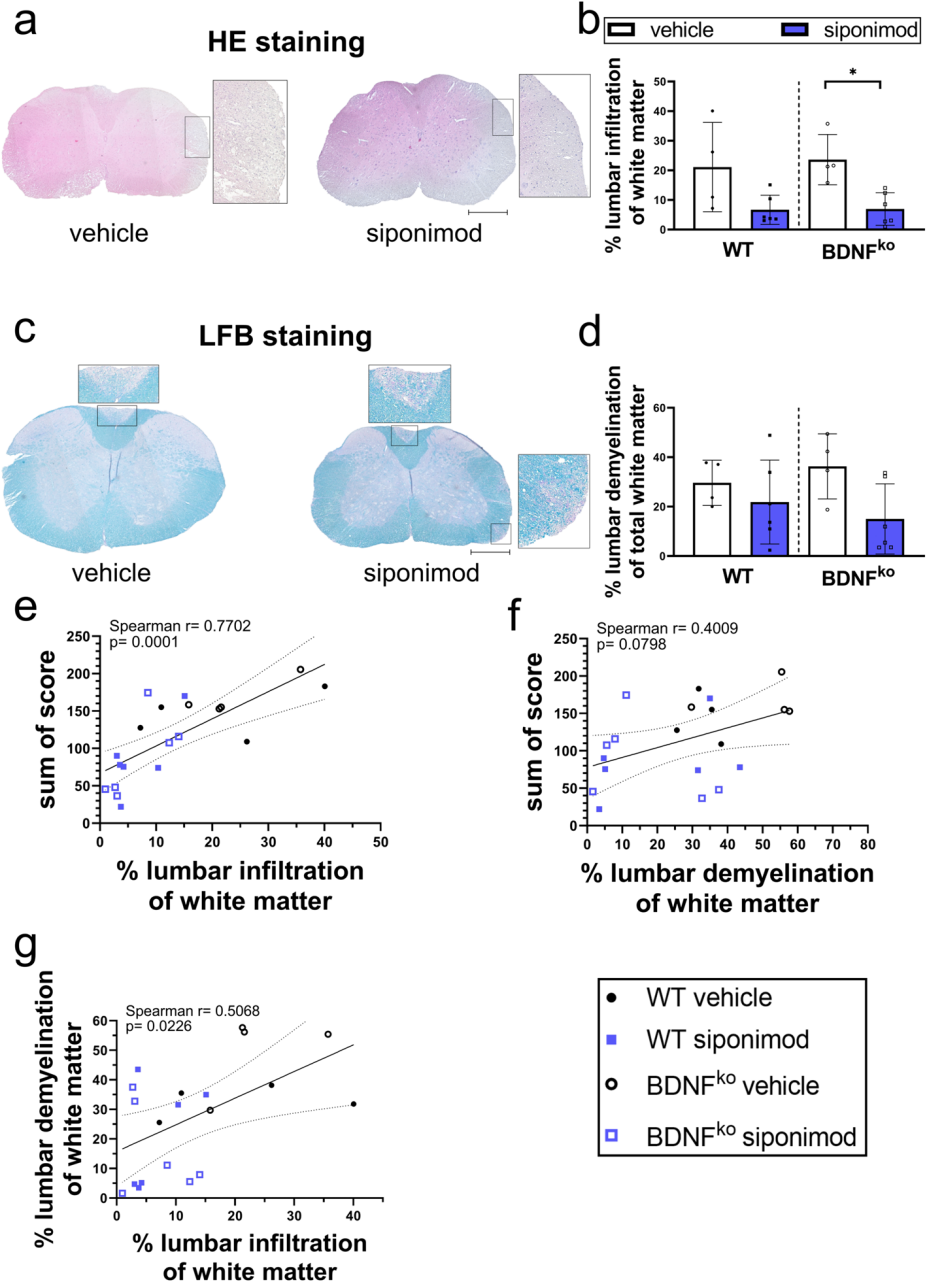
Siponimod treatment reduces lymphocyte infiltration of white matter in lumbar spinal cord. (**a**) Represenative hematoxylin and eosin (HE) images of spinal cord sections. (**b**) BDNF^ko^ EAE mice showed less lumbar lymphocyte infiltration when administered with 0.45 µg siponimod daily, which is also suggested for WT mice. (**c**) Represenative LFB images of spinal cord sections. (**d**) There is no significant reduction of demyelination for lumbar white matter caused by 0.45 µg/day siponimod independent of BDNF. (**e**) High disease activity strongly correlated with higher lymphocyte infiltration in respective mice. (**f**) Higher degress of demyelination for lumbar white matter area seemed to be linked with sum of scores, yet missing significance narrowly. (**g**) Mice with more infiltration showed a trend towards more demyelination in lumbar spinal cord, missing significance. Lumbar sections of spinal cord were analyzed in quadruplicates for each mouse. WT vehicle: n = 4, WT siponimod: n = 6, BDNF^ko^ vehicle: n = 4, BDNF^ko^ siponimod: n = 6. Two mice without EAE symptoms (WT vehicle: n = 2) and two mice with premature exitus (BDNF^ko^ vehicle: n = 2) were excluded. Data are shown as mean ± SEM. Infiltration and demyelination data (**b**, **d**) were analyzed for normality with Shapiro–Wilk test and tested for significance with Kruskal–Wallis test with post-hoc analysis using Dunn`s multiple comparisons test. Correlation analysis (**e**–**g**) was performed using Spearman correlation and 95% confidence bands are depicted. Significances are depicted as *p < 0.05. Scale bars: 500 µm. *HE* hematoxylin & eosin staining, *LFB* Luxol’s Fast Blue staining.

To understand whether clinical effects were also corroborated histologically, we investigated immune cell infiltration and demyelination of cervical, thoracic and lumbar spinal cord. For vehicle groups, higher percentages of lumbar (Fig. 4b, 21.1–23.6% (± 7.4–4.3%)) white matter were infiltrated compared to cervical parts (12.1–12.2% (± 3.3–4.1%); Fig. S3a). Analysis of lumbar spinal cord revealed a trend towards reduction of immune cell infiltration following siponimod treatment for WT mice and a significant decrease for BDNF^ko^ mice (Fig. 4a,b). Likewise, demyelination trended to be reduced in siponimod treated mice in lumbar white matter. WT control mice and BDNF^ko^ mice displayed 29.6 ± 4.6% and 36.3 ± 6.6% of demyelination in lumbar spinal cord, respectively. In contrast, siponimod treated mice showed 21.8 ± 6.9% demyelination for WT and 15 ± 5.8% for BDNF^ko^, reducing it by more than 50% in the latter case (Fig. 4c,d). For vehicle groups, the demyelinated white matter area increased towards caudal regions, being lowest in cervical area (21.7–26.5% (± 4–8.6%); Fig. S3b) and highest in lumbar area (Fig. 4d, 29.6–36.3% (± 4.5–6.6%)). There was a strong correlation of lumbar immune cell infiltration with disability as assessed using sum of scores (r = 0.77, p < 0.0001; Fig. 4e). Demyelination of white matter did not significantly correlate with sum of scores in mice (p = 0.08; Fig. 4f). However, immune cell infiltration strongly correlated with demyelination in lumbar spinal cord (r = 0.5068, p = 0.0226; Fig. 4g), as well as for cervical and thoracic parts (Fig. S3c).

We also investigated alterations of immune cell subpopulations in blood, spleen, and lymph nodes via flow cytometry. Siponimod 0.45 µg/day had no effect on ratio of CD3-positive T cells, CD19-positive B cells and CD11b/F4/80-positive macrophages (Fig. S4). Th cells and their Th1, Th17 and Treg subpopulations showed no regulation in frequencies (Fig. S4). We saw decreased frequencies of Tc cells in spleen and lymph nodes for BDNF^ko^ mice compared to WT mice (p = 0.0237 for spleen, p = 0.0125 for lymph nodes; Fig. S4c). Siponimod increased Tc1 ratio in lymph nodes (p = 0.0268; Fig. S4i) and reduced Tc17 frequencies in spleen (p = 0.0474; Fig. S4j).

### Modulation of EAE splenocyte induced neurotoxicity by siponimod

**Figure 5:**
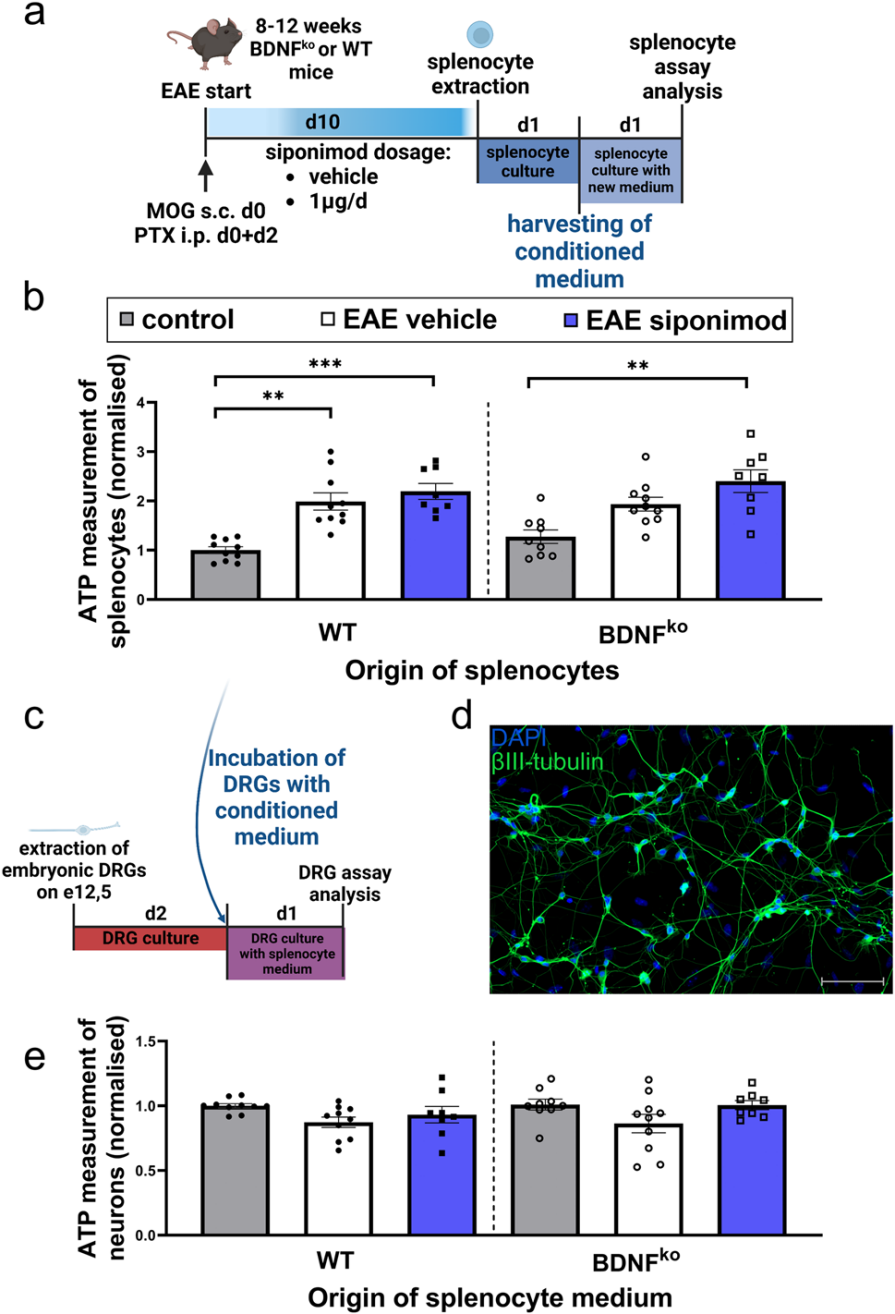
Analysis of neurotoxicity induced by EAE conditioned media of splenocytes. (**a**) Splenocytes from mice 10 d post immunization were cultured and conditioned media were harvested 24 h after medium change. Created with BioRender.com. (**b**) MOG-immunisation and siponimod treatment led to a BDNF-independent increase of splenocyte viability, using an ATP-Glo assay. (**c**) Embryonal DRGs were incubated for 24 h with conditioned media of splenocytes. (**d**) Represenative βIII-tubulin staining of DRG networks. (**e**) Neurons incubated with conditioned media of EAE splenocytes show a trend towards reduction of viability, measured by using an ATP-Glo assay, which can be halted by prior siponimod administration of EAE mice independent from BDNF. For viability (**b**, **e**), n = 5 experiments with n = 2 mice in each group are presented. WT control: n = 10, WT EAE vehicle: n = 10, WT EAE siponimod: n = 8, BDNF^ko^ control: n = 9, BDNF^ko^ EAE vehicle: n = 10, BDNF^ko^ EAE siponimod: n = 8. For splenocytes (**b**), 3 to 4 replicates and for neurons (**e**), 8–12 replicates are presented. Data were normalized to their respective WT control group and shown as mean ± SEM. Data (**b**, **e**) were tested for normality with Shapiro–Wilk test and tested for significance with Kruskal–Wallis test with post-hoc analysis using Dunn’s multiple comparisons test. Significances are depicted as **p < 0.01, ***p < 0.001. Scale bar: 100 µm. *EAE* experimental autoimmune encephalomyelitis, *DRG* dorsal root ganglion cell.

To investigate effects of siponimod on the interaction between neurons and immune cells in absence of BDNF, we performed in vitro experiments using dorsal root ganglion cells (DRGs) and splenocytes from control, EAE vehicle and EAE siponimod treated mice. First, we assessed viability of splenocytes by measuring ATP quantity (Fig. 5a). Splenocyte viability was significantly more pronounced in WT EAE mice compared to WT control mice (p < 0.01; Fig. 5b). Siponimod treated splenocytes had similar viability compared to vehicle treated EAE mice (ns). Splenocytes from BDNF^ko^ mice showed the same pattern as WT splenocytes. Thus, we suggest that both EAE induction and siponimod treatment activates splenocytes, increasing their viability.

We then incubated DRGs with splenocyte conditioned media for 24 h and then assessed neuronal viability (Fig. 5c). BetaIII tubulin staining showed the formation of neuronal networks for DRG cells (Fig. 5d). In this setup, we did not see significant changes, although there was a small trend suggesting that conditioned media from WT EAE splenocytes has harmful effects on DRGs (Fig. 5e). Siponimod administration seemed to have an beneficial influence on affected neurons, as viability seemed to improve for siponimod groups, independent of BDNF (all ns).

### Siponimod shows BDNF-dependence regarding transcription of chemokines

**Figure 6:**
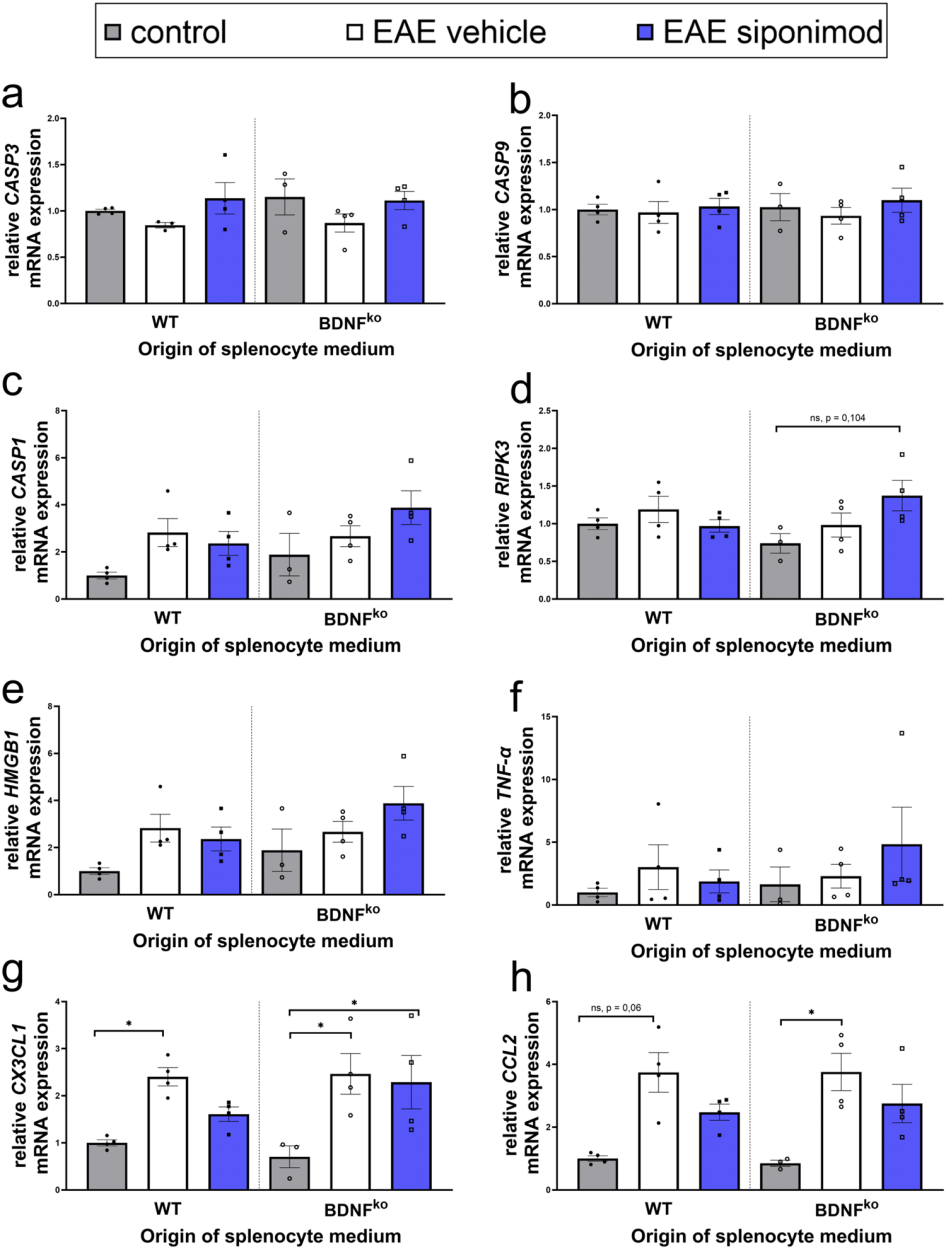
Siponimod treatment BDNF-dependently reduces neuronal expression of CX3CL1. Investigation of mRNA expression of neurons from Fig. 5c. (**a**, **b**, **f**) Expression of apoptosis markers CASP3 and CASP9 and TNF-a were not affected. (**c**) Expression of the pyroptosis activator CASP1 was not regulated significantly, showing trends of increase in EAE groups. (**d**) The necroptosis marker RIPK3, and (**e**) the necrosis marker HMGB1 show trends toward elevation in neurons in contact with splenocyte medium from BDNF^ko^ mice following siponimod treatment. (**g**, **h**) Expression of CX3CL1 and CCL2 was enhanced following incubation with EAE conditioned media. For the former, this increase was BDNF-dependently alleviated following siponimod administration. n = 2 experiments with n = 2 mice in each group in duplicates were performed. WT control: n = 4, WT EAE vehicle: n = 4, WT EAE siponimod: n = 4, BDNF^ko^ control: n = 3, BDNF^ko^ EAE vehicle: n = 4, BDNF^ko^ EAE siponimod: n = 4. Data of one mouse in BDNF^ko^ control was contaminated. Means of technical replicates were normalized to their respective WT control group and shown as mean ± SEM. Data were tested for normality with Shapiro–Wilk test and tested for significance with Kruskal–Wallis test with post-hoc analysis using Dunn’s multiple comparisons test. Significances are depicted as *p < 0.05.

We analysed the transcriptome of DRGs to understand the underlying mechanisms of neuronal loss following incubation with EAE conditioned media in vitro. We analysed apoptosis, which proceeds immunologically silently^32^. Neurons in contact with conditioned medium from EAE mice did not show genetic regulation of the most prominent apoptosis markers CASP3, the main effector caspase (Fig. 6a), and CASP9, which is an initiator caspase (Fig. 6b). Furthermore, we saw no effects on Caspase-3/-7 activity in these neurons (Fig. S5a). Additionally, we saw no differences in the transcription of cytochrome c, which is a mitochondrial protein of the respiratory chain that is ejected into the cytosol during apoptosis, the protein BAD, which is a representative of the BCL-2 family that modulates apoptosis, and Beclin 1, which serves as an autophagy signal and regulates apoptosis^33^ (Fig. S6a–d). Therefore, transcription of typical apoptosis markers as well as Caspase-3/-7 activity are not affected by early interactions between neurons and lymphocytes.

We extended the analysis towards alternative death pathways and investigated pyroptosis, an inflammatory programmed cell death driven by Caspase 1. DRGs exposed to EAE medium had a slight, insignificant increase for the transcription of CASP1 in relation to control (Fig. 6c). Supernatant of siponimod treated WT splenocytes seemed to lower the neuronal CASP1 transcription only insignificantly. There was an inverse effect for BDNF^ko^, which rather showed an insignificant increase of CASP1 expression for siponimod groups.

Another alternative death pathway is necroptosis. Like pyroptosis, necroptosis is activated by Pathogen-associated molecular patterns (PAMPs) and the inflammatory protein TNF-α, which activates receptor-interacting serine/threonine protein kinases 1 and 3 (RIPK1/RIKP3). RIPK3 transcription showed a trend towards elevation in neurons in the BDNF^ko^ group despite siponimod administration (p = 0.1 for BNDF^ko^ ctrl vs. BDNF^ko^ EAE sip; Fig. 6d), but not for WT, indicating that BDNF-deficiency in key immune cells could have an effect on necroptosis. This is of special note, as BDNF shares the downstream target ERK1/2/MAPK with the necroptotic pathway^20,34^. This was in line with regulation of HMGB1, a marker for necrosis which further boosts inflammation (ns; Fig. 6e). TNF-α, which is a key inflammatory marker and initiator of necroptosis and pyroptosis, showed the same trends as RIPK3 (ns; Fig. 6f).

We analysed the genetic regulation of chemotactic markers CCL2 and CX3CL1. Neurons incubated with EAE splenocyte medium showed a significant activation of CX3CL1 transcription (p = 0.05 for WT, p = 0.0224 for BDNF^ko^; Fig. 6g). Whilst CX3CL1 transcription was still significantly elevated for BDNF^ko^ group despite siponimod treatment (p = 0.0426), we did not see the same significant increase for neurons in contact with siponimod-treated EAE splenocytes of WT mice (ns). This was similar for the regulation of CCL2, which showed an almost 4-fold transcription increase in neurons affected by EAE medium (p = 0.06 (ns) for WT, p = 0.0358 for BDNF^ko^; Fig. 6h). Prior siponimod administration resulted in a non-significant, and less distinct, increase of CCL2 transcription. Thus, we deduce that siponimod might not be able to regulate the transcription of neuronal chemokines in BDNF-deficient environments. Incidentally, siponimod did not have any significant effects on the transcription of neuronal BDNF or its main receptor trkB (Fig. S6e).

In summary, siponimod showed no BDNF-dependency in vivo in EAE but possible BDNF-dependency regarding the modulation of neuronal inflammation in vitro.

## Discussion

Multiple sclerosis progression remains a challenge for treating physicians since most medications effective in the treatment of RRMS yield ineffective results. The EXPAND study highlighted the S1PR-1 and -5 modulator siponimod as a putative tool in tackling SPMS^6^. Nevertheless, it remains unclear how siponimod might mediate neuroprotective effects when most other medications, including its predecessor fingolimod, failed. In this study, we investigated the importance of BDNF for the effects of siponimod in vivo and in vitro. Siponimod administered in chronic experimental autoimmune encephalomyelitis (EAE) ameliorated clinical signs of EAE. The suboptimal dosage of 0.45 µg/day had a marginal effect, which was not BDNF-dependent. We saw a reduction of immune cell infiltration into lumbar white matter area (Fig. 4b) for this siponimod dosage, and documented correlations between demyelination and immune cell infiltration into white matter (Fig. 4g). Higher siponimod dosages led to a retention of autoreactive immune cell populations in secondary lymphoid organs. In vitro, there were BDNF-dependent effects on transcription of certain inflammatory markers produced by neurons.

Siponimod reduces EAE scores due to its pleiotropic effects on lymphocyte egress, circulation and chemoattraction^3,5^. Furthermore, siponimod reduces microglial activation and induces axonal remyelination by inducing OPCs^13,14^. The effects of siponimod in EAE models have been displayed with various dosages and application methods^3,5,30^. Our aim was to mimic the daily oral intake of 1–2 mg/day by human beings, which approximates the dosage of 4–8 µg/day in mice^8,35^. Human equivalent dosages halted EAE disease activity completely, while lower dosages elicited alleviating effects, even for oral administration of the suboptimal dosage of 0.45 µg/day^3,30^.

In our study, we could define more closely how siponimod affects lymphocyte populations. As was reported previously by Gentile et al.^3^, who used intracerebroventricular administration, we saw no effects on peripheral lymphocyte count. Gergely et al. showed data which indeed shows reduction of absolute lymphocyte count, however, for human beings with dosages > 1 mg/day^5^. For human equivalent dosages, we saw an increase of B cell frequencies in lymph nodes, whereas Tc and Th cells were reduced in secondary lymphatic organs as shown previously using different mouse models^30^. Tregs exhibited a profound decrease in blood, and an increase in lymph nodes. Moreover, Treg frequencies negatively correlated with Th1 and Th17 frequencies in spleen. We postulate that the different effects of siponimod on Tregs as well as Th1 and Th17 in lymphoid organs may explain part of its beneficial immunomodulatory effects. This shift could be explained by the chemokine CCR7, which can be seen as the antagonist of S1P and holds back lymphocytes in lymph organs^36^. Thus, siponimod effects are more pronounced on CCR7-positive Th1 and Th17 cells than CCR7-negative Tregs. We assume that this shift of autoreactive to protective lymphocytes may play a role in the neuroprotective effect of siponimod.

Immunohistochemistry of spinal cord showed that siponimod reduces immune cell infiltration in lumbar white matter and suggests a reduction of demyelination, consistent with previous findings^11^. Of note, siponimod also reduces the size of meningeal ectopic lymphoid tissue^30^. Both these histological parameters are linked to disease severity but were not affected in the BDNF^ko^ model used here. The generally accepted model proposes that lymphocyte infiltration causes demyelination, which, in turn, affects the motor functions of mice. This can explain why EAE symptoms predominantly begin in hind legs, as lumbar spinal cord shows more demyelination and leukocyte infiltration than cervical spinal cord. However, Frezel et al. argues lymphocyte infiltration to be a secondary effect, as neuronal production of the stress-associated transcription factor ATF3 precedes T cell infiltration^37^. Thus, we wanted to characterize the influence of infiltrating lymphocytes on neurons.

Effects of fingolimod are BDNF-dependent in hippocampal cultures, since treatment with BDNF-scavenging trkB receptor bodies diminishes fingolimod effects on neuronal dendrite growth and activation, questioning whether there might be an in vivo correlate^20^. We could show that siponimod reduces EAE scores and improves general well-being even in BDNF^ko^ mice. Furthermore, we could show that EAE and siponimod administration affects splenocyte viability, although we could not clearly show the effect of these splenocytes on embryonal neurons. It is important to note that our Cre-loxP-model supported BDNF-deficiency on T cells and microglia/macrophages. Although BDNF is also produced by neurons and glia such as astrocytes, activated immune cells are the major source of BDNF in active MS lesions^23,38^. Kerschensteiner et al. could show in vitro that this BDNF-secretion of immune cells provides protective effects on neuronal survival^39^, giving infiltrating immune cells a context-sensitive neuroprotective role. As we saw no aggravation of EAE symptoms in BDNF^ko^ mice, autoreactive effects by infiltrating immune cells seem to be outdoing protective effects of immunological BDNF, relegating immunological BDNF to a secondary role; at least in the model used here. BDNF^ko^ in further cell types could pertain a more severe EAE progress; however, more severe BDNF-deficiency also leads to aggressive behaviour, memory deficits and even early death in mice^40,41^. Therefore, our cell specific BDNF^ko^ model provided an ethically acceptable level of genetic alteration, as mice did not show phenotypic abnormalities. There have been concerns about the specificity of lysM- Cre for macrophages/microglia^42^. Linker et al. were able to show that targeting lysM does not incur BDNF reduction in brain tissue^23^.

The lack of BDNF in key immune cells did not show an effect on the transcription of initiation markers for pyroptosis and necroptosis. These death pathways share the activation by PAMPs, DAMPs and TNF-α, and both serve, in contrast to typical apoptosis, as a potent amplifier of inflammation^34,43^. Both pathways have an executing protein complex. Pyroptosis is driven by inflammasomes containing CASP1, ASC and sensor markers such as NLRP1 or NLRP3. This leads to Gasdermin D-mediated pyroptotic bodies that cause neuroinflammation and CASP6-mediated demyelination^44,45^. MS medications such as cladribine and IFN-β have been shown to regulate pyroptosis^43^, which is unaffected by siponimod with the methods used here. In the case of necroptosis, necrosomes formed by RIPK1/RIPK3 activate the pro-necroptotic protein MLKL, which causes a caspase-8-independent cell death. Necroptosis has been described as a backup plan for cell death, as apoptosis and pyroptosis markers are capable of inhibiting necroptosis^32^. Additionally, RIPK1/3 leads to inflammation by a prolonged activation of ERK1/2/MAPK pathway^34^. This is noteworthy as ERK1/2 and akt phoshorylation are a target of BDNF as well and play a major role in counteracting cell death^20^. However, necroptosis leads to a prolonged activation of this pathway which turns this naturally protective and remyelinating pathway deleterious^46^. We hypothesize that ERK1/2 remains unnaturally activated by necroptosis as BDNF decreases. This might explain why BDNF-deficiency does not have a detrimental effect on EAE progress in our model, as the protective influence of the BDNF-ERK1/2 pathway might have been hijacked for inflammation by necroptosis, even in BDNF-rich environments. Yet, we cannot clearly show that siponimod prevents necroptosis and it remains an interesting topic for further research to investigate the link between BDNF and necroptosis.

Although a lack of BDNF in key immunomodulatory cells alone is not stressful for neurons, hinted by preserving their viability (Fig. 5e), it becomes stressful in neuroinflammatory environments that cannot be alleviated by siponimod, indicated by the increased transcription of the chemokines CX3CL1 and CCL2, produced by neurons upon inflammation which act on immune cells, especially microglia.

CX3CL1, also called fractalkine, is a neuronal transmembrane protein that can be cleaved to act as a soluble chemokine^47^. It attracts CX3CR1 expressing immune cells like microglia and T cells, maintaining inflammatory states^47^. However, the infiltrating CX3CR1-positive microglia elicit neuroprotection through phagocytosis and through ERK1/2 and akt signalling^48^, sharing the same pathway as BDNF. As BDNF and CX3CL1 share the same downstream target, it might be assumed that BDNF-deficiency is compensated by enhanced CX3CL1 expression. Above, we already discussed that a prolonged activation of the ERK1/2 pathway boosts inflammation. CCL2, also known as MCP-2, is a chemokine that mediates part of the chemoattractive effects of S1P. Upon binding S1PR-2, S1P enhances CCL2 secretion in neurons, which leads to an attraction and activation of CCR2 positive immune cells^49,50^. Additionally, CCR2 activated microglia are stimulated to express P2X7 receptor, which in turn leads to secretion of microglial BDNF^50^. This S1PR-2-CCL2-BDNF pathway has been argued to mediate neuroinflammation and demyelination. As we saw CCL2 modulation with a S1PR-1 and -5 modulator, CCL2 might also be modulated by those subunits. However, the lack of microglial BDNF in our BDNF^ko^ mice and the CCL2 modulation did not reveal any particularly deleterious effects. We can support the hypothesis that the synthesis of both these neuronal chemokines increases during neuroinflammation but normalizes due to the S1PR-1 and -5 modulator siponimod, which also leads to less lymphocyte infiltration. As these chemokines remain elevated despite siponimod upon lack of BDNF in immune cells, we assume that protective effects of siponimod are mitigated in part by BDNF-deficiency.

In summary, we showed that treatment with siponimod ameliorates clinical signs of EAE. In vivo, clinical effects were not mediated by the presence of BDNF in immune cells. Siponimod, however, showed partial BDNF-dependency for modulation of key chemoattractive pathways. Further research may uncover the role and dependencies of BDNF in inflammatory pathways and alternative death pathways, presenting new targets for immune therapies.

### Supplementary Information

Supplementary Information.

## Data Availability

All data are available from the corresponding author SF upon reasonable request.

## Abbreviations

MS: Multiple sclerosis
S1PR: Sphingosine-1-phosphate receptor
CNS: Central nervous system
BDNF: Brain derived neurotrophic factor
EAE: Experimental autoimmune encephalomyelitis
WT: Wildtype
BDNF^ko^: Partial BDNF knock out
HE: Hematoxylin & eosin
LFB: Luxol’s® Fast Blue
DRG: Dorsal root ganglion
RRMS: Relapsing remitting multiple sclerosis
BBB: Blood–brain barrier
GM: Grey matter
DMT: Disease modifying therapy
SPMS: Secondary progressive multiple sclerosis
S1P: Sphingosine-1-phosphate
ERK: Extracellular signal-regulated kinase
MAPK: Mitogen activated protein kinase
MOG: Myelin oligodendrocyte glycoprotein
PTX: Pertussis toxin
Th cell: T-Helper cell
Tc cell: Cytotoxic T cell
Tregs: T regulatory cells
RIKP3: Receptor-interacting serine/threonine protein kinase 3
