# Supplementary material for: Siponimod treatment response shows partial BDNF dependency in multiple sclerosis models
Source: Sci Rep. 2024 Aug 1;14:17823. doi: 10.1038/s41598-024-68715-x (PMC11294562; doi:10.1038/s41598-024-68715-x)
Supplement: Supplementary file 1 — Supplementary Information. [file 41598_2024_68715_MOESM1_ESM.docx]

**SUPPLEMENT**

# Siponimod Treatment Response shows partial BDNF Dependency in Multiple Sclerosis Models

Hasan Hüseyin Hendek ^1^, Alina Blusch ^1^, Neele Heitmann ^1^, Sarah Oberhagemann ^1^, Seray Demir ^1^, Xiomara Pedreiturria ^1^, Ralf Gold ^1^, Simon Faissner ^1^

^1^ Department of Neurology, Ruhr-University Bochum, St. Josef-Hospital, Bochum, Germany

Corresponding author

Prof. Dr. Simon Faissner, MD

Department of Neurology

Ruhr-University Bochum, St. Josef-Hospital

Gudrunstr. 56, 44791 Bochum, Germany

Tel: +49-234-5092411; Fax: +49-234-5092740

Email: simon.faissner@rub.de

# SUPPLEMENTARY TABLES

## Table S1: Antibodies used for flow cytometry

| **Target** | **Fluorochrome** | **Dilution** | **Clone** | **Reference number** | **Vendor** |
| --- | --- | --- | --- | --- | --- |
| CD19 | APCCy7 | 1:200 | 1D3 | 561 737 | BD Biosciences, Franklin Lakes, NJ, USA |
| CD11b | FITC | 1:200 | M1/70 | 101 205 | Biolegend, San Diego, CA, USA |
| CD25 | APC | 1:200 | PC61.5 | 17-0251-81A | Invitrogen, Waltham, MA, USA |
| FoxP3 | PE | 1:200 | FJK16S | 12-5773-82 | eBiosciences, Santa Clara, CA, USA |
| CD45 | BV650 | 1:200 | 30-F11 | 103 151 | Biolegend, San Diego, CA, USA |
| IFN-γ | BV605 | 1:100 | XMG1.2 | 505 840 | Biolegend, San Diego, CA, USA |
| F4/80 | BV421 | 1:50 | BM8 | 123 132 | Biolegend, San Diego, CA, USA |
| IL-17A | PE/Dazzle 594 | 1:50 | TC11-18H | 506 937 | Biolegend, San Diego, CA, USA |
| CD3 | Alexa Fluor 700 | 1:100 | 17A2 | 100 215 | Biolegend, San Diego, CA, USA |
| CD4 | BV785 | 1:200 | RM4-5 | 100 552 | Biolegend, San Diego, CA, USA |
| CD8a | PerCP/ Cyanine5.5 | 1:200 | 53-6.7 | 100 734 | Biolegend, San Diego, CA, USA |
| CD16/CD32 | - | 1:200 | 93 | 14-0161-81-A | eBiosciences, Santa Clara, CA, USA |
| ZombieAqua Fixable Viability Kit |  | 1:1500 |  | 423 101 | Biolegend, San Diego, CA, USA |
| DMSO | - | 1:1500 | - | 77 144 | Biolegend, San Diego, CA, USA |

## Table S2: Antibodies used for immunohistochemistry

| **Antibody** | **Dilution** | **Species** | **Reference number** | **Vendor** |
| --- | --- | --- | --- | --- |
| anti-βIII-tubulin | 1:7500 | rabbit | T2200-200UL | Sigma Aldrich GmbH, Steinheim, Germany |
| Alexa Fluor® Plus 488 anti-rabbit IgG (H+L) highly cross-adsorbed | 1:1000 | goat | A32731 | Invitrogen by Thermo Fisher Scientific, Eugene, OR, USA |
| Anti-Iba1 | 1:200 | chicken | 234 009 | SynaticSystems GmbH, Goettingen, Germany |
| Cy3-fluorochrome anti-chicken | 1:100 | donkey | AP194C | MerckMillipore, Darmstadt, Germany |
| anti-mouse CD4 | 1:100 | Rat | 14-9766-80 | Thermo Fisher Scientific, Eugene, OR, USA |
| Alexa Fluor® 488 anti-rat | 1:500 | goat | A-11006 | Invitrogen by Thermo Fisher Scientific, Eugene, OR, USA |
| anti-BDNF | 1:100 | rabbit | AB1534 | MerckMillipore, Darmstadt, Germany |
| Cy5-fluorochrome anti-rabbit | 1:1000 | goat |  | MerckMillipore, Darmstadt, Germany |
| DAPI- Fluoromount | - | - | 0100-20 | SouthernBiotech by Biozol, Eching, Germany |

## Table S3: Medium for neuronal culture

| **Name** | **Volume and concentration** | **Vendor** |
| --- | --- | --- |
| Neurobasal medium | - | Thermo Fisher Scientific, Schwerte, Germany |
| B27- supplement | 2 % | Thermo Fisher Scientific, Schwerte, Germany |
| horse serum | 2 % | Thermo Fisher Scientific, Schwerte, Germany |
| L-glutamine | 1 % | Thermo Fisher Scientific, Schwerte, Germany |
| Penicillin/streptomycin | 0.5 % | Thermo Fisher Scientific, Schwerte, Germany |
| NGF | 0.1 % (only in growth) | Sigma Aldrich GmbH, Steinheim, Germany |

## Table S4: Medium for splenocyte culture

| **Name** | **Volume and concentration** | **Vendor** |
| --- | --- | --- |
| RPMI 1640 medium | - | gibco by Thermo Fisher Scientific, Schwerte, Germany |
| FCS | 10% | Sigma Aldrich GmbH, Steinheim, germany |
| Sodium pyruvate | 1% | gibco by Thermo Fisher Scientific, Schwerte, Germany |
| Glutamax | 1% | Thermo Fisher Scientific, Schwerte, Germany |
| Penicillin/Streptomycin | 1% | Thermo Fisher Scientific, Schwerte, Germany |
| HEPES | 1% | gibco by Thermo Fisher Scientific, Schwerte, Germany |
| 2-Mercaptoethanol | 0,1% | gibco by Thermo Fisher Scientific, Schwerte, Germany |
| MOG_35-55_ | 10-25µg/ml | Genosphere Biotechnologies, Paris, France |

## Table S5: Primer sequences

| **Primer name** | **Sequence** | **Efficiency** | **Vendor** |
| --- | --- | --- | --- |
| Caspase 3  (CASP3) | F: 5´- GCCCAGGAAGCTATTGACAAC -3´  R: 5´- CAGTCAAGAGTGGGCCCATC -3´ | 80% | Microsynth Seqlab GmbH, Göttingen, Germany |
| Caspase 9  (CASP9) | F: 5´- CTGCAGGACACACAGGAGAG -3´  R: 5´- CAGGATTCCGGGCTAAGACC -3´ | 95% | Microsynth Seqlab GmbH, Göttingen, Germany |
| Caspase 1  (CASP1) | F: 5´- TCAGGGGCTCACTTTTCATTGA -3´  R: 5´- GGTCACCCTATCAGCAGTGG -3´ | 92,3% | Microsynth Seqlab GmbH, Göttingen, Germany |
| Receptor Interacting Serine/Threonine Kinase 3  (RIPK3) | F: 5´- GCACCACCAAGAACTACTGC -3´  R: 5´- TGGCACACTTCAGGCTCTTG -3´ | 104% | Microsynth Seqlab GmbH, Göttingen, Germany |
| High-Mobility-Group-Protein B1 (HMGB1) | F: 5´- GGCTCTTTCCCATTAACAACCC -3´  R: 5´- CTGGTTCCCTCCTTTTGCCA -3´ | 105,6% | Microsynth Seqlab GmbH, Göttingen, Germany |
| Tumor necrosis factor α  (TNFα) | F: 5´- AAA TGG CCT CCC TCT CAT CAG -3´  R: 5´- GTC ACT CGA ATT TTG AGA AGA TGA TC -3´ | 117,7% | Microsynth Seqlab GmbH, Göttingen, Germany |
| C-X3-C Motif Chemokine Ligand 1 (= fractalkine)  (CX3CL1) | F: 5´- GCAACCCCACCCCTTATCAA -3´  R: 5´- GCACTCTCAAGCCACTCCTT -3´ | 83% | Microsynth Seqlab GmbH, Göttingen, Germany |
| CC-chemokine ligand 2 (= MCP1)  (CCL2) | F: 5´- GTCTGTGCTGACCCCAAGAA -3´  R: 5´- CGGGTCAACTTCACATTCAAAGG -3´ | 94% | Microsynth Seqlab GmbH, Göttingen, Germany |
| Beta-actin | F: 5´- CATGTTTGAGACCTTCAACACCCC-3´  R: 5´- GCCATCTCCTGCTCAAAGTCTAG -3´ | - | Microsynth Seqlab GmbH, Göttingen, Germany |
| 18S | F: 5´- GTAACCCGTTGAACCCCATT -3´  R: 5´- CCATCCAATCGGTAGTAG C -3´ | - | Microsynth Seqlab GmbH, Göttingen, Germany |
| Cytochrome c  (CYCS) | F: 5´- TGCAGAAAGGGGACAGTCTC -3´  R: 5´- TGAACTAGCCACCTACAAAGCA -3´ | 117% | Microsynth Seqlab GmbH, Göttingen, Germany |

| BCL2 Associated Agonist Of Cell Death (BAD) | F: 5´- ATATTGGGTGTGGGCGGAAG -3´  R: 5´- GGGACTCAAGCTGTACGTCA -3´ | 111% | Microsynth Seqlab GmbH, Göttingen, Germany |
| --- | --- | --- | --- |
| Apoptosis regulator B-cell lymphoma 2  (BCL2) | F: 5´- CAGCTGGCTCCCTTCATGAA -3´  R: 5´- GAGAACCCCTGTCTCCAAAGG -3´ | 111% | Microsynth Seqlab GmbH, Göttingen, Germany |
| Beclin1 | F: 5´- GAGGAAGGCTTGCCATGAGA -3´  R: 5´-TGAGCCACCTATCTGACCCA -3´ | 97% | Microsynth Seqlab GmbH, Göttingen, Germany |
| Brain derived neurotrophic factor  (BDNF | F: 5´- ACTGCAGTGGACATGTCTGG -3´  R: 5´- CTGCAGCCTTCCTTGGTGTA -3´ | 102,4% | Microsynth Seqlab GmbH, Göttingen, Germany |
| Tropomyosin receptor kinase B  (trkB) | F: 5´- AGGAAATTCACCACCGAGAGC -3´  R: 5´- GACTCTTCCCTGGGTGATGC -3´ | 103,2% | Microsynth Seqlab GmbH, Göttingen, Germany |
| ATP synthase membrane subunit f  (ATP5j2) | F: 5´- CCGCTGAAGGAGAAGAAGCT -3´  R: 5´- GCCTTTCCGAACGTTGATGT -3´ | 108,1% | Microsynth Seqlab GmbH, Göttingen, Germany |


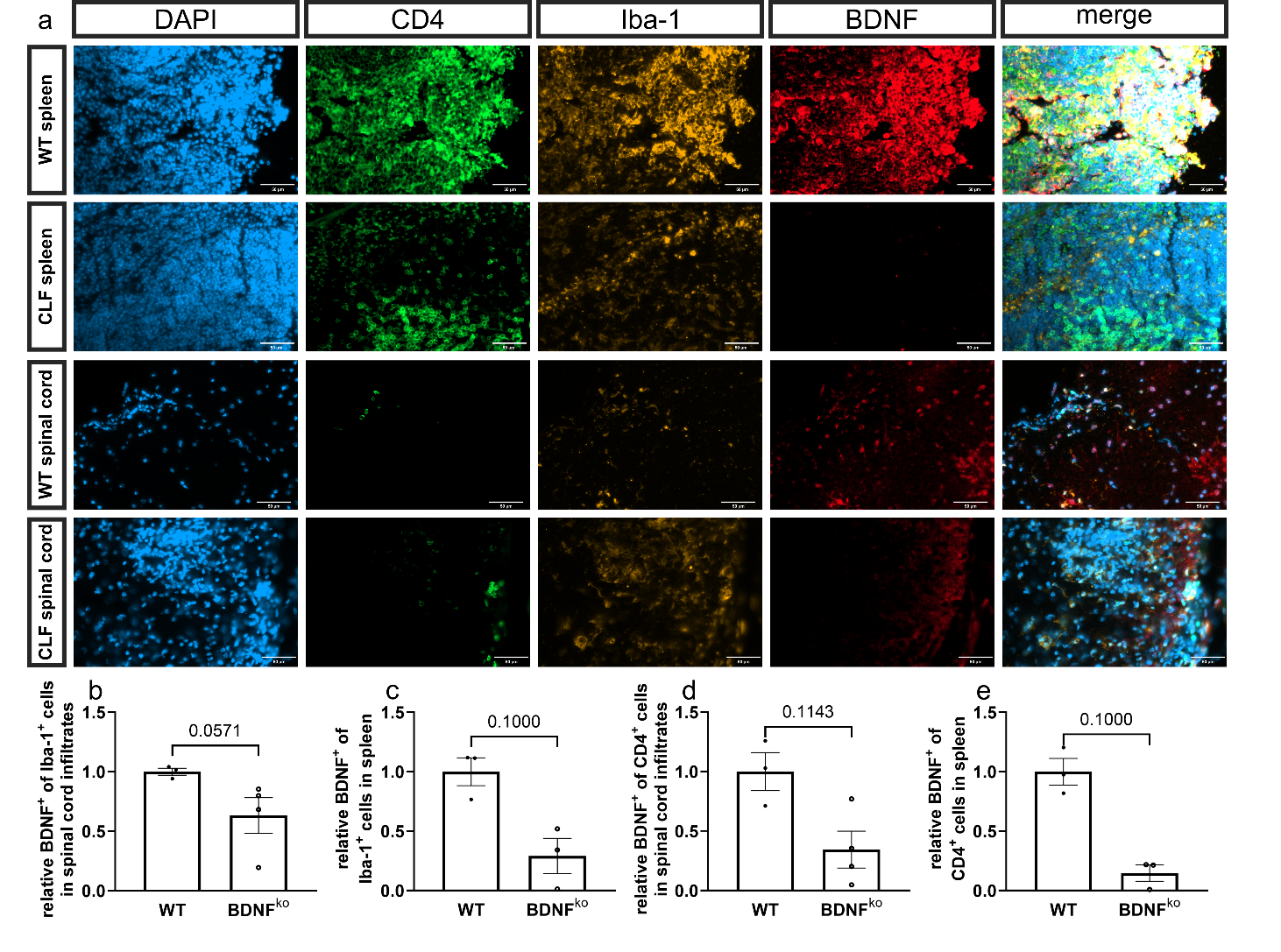


**Figure S1: BDNF-positive leukocytes are diminished both in spleen and in spinal cord infiltrates for BDNF^ko^ mice compared to WT.** a) Representative images of the immunohistochemical staining of paraffin embedded spleen and spinal cord from WT and BDNF^ko^ mice. b)-e) Graphical representation of relative BDNF immunopositive cells of CD4^+^ T helper cells or IBA1^+^ macrophages/microglia in b)/d) spinal cord crossections and c)/e) spleen sections. The number of BDNF^+^ cells of the respective cell type was calculated and normalized to the mean BDNF^+^ cells of the C57Bl/6 (WT) mice. b-c) There is a tendency of a relative reduction of BDNF in IBA1^+^ monoctyes/macrophages in BDNF^ko^ mice both for IBA^+^ cells in spleen and in spinal cord. d-e) BDNF expression seems to be hindered in Th cells with BDNF^ko^ mice, both for Th cells in spleen and in spinal cord. For b) and d): WT: n=3, BDNF^ko^: n=4. For c) and e): WT: n=3, BDNF^ko^: n=3. Data are shown as mean±SEM. Data were tested for normality with Shapiro-Wilk test and tested for significance with Mann-Whitney test. Scale bars in a) represent 50 µm.


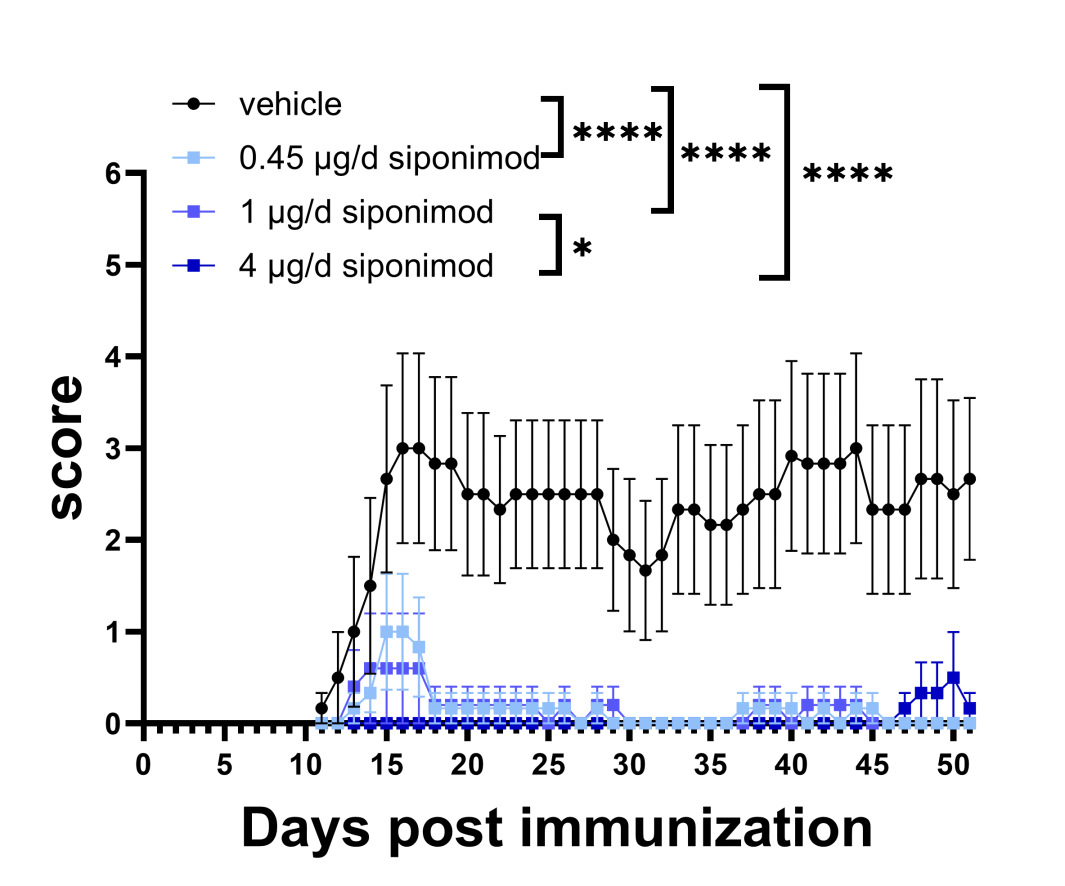
 **Figure S2: Data of Figure 1 without outlier.** Clinical scores were significantly lowered for all siponimod dosages. . n=6 for each group, except n=5 for 1 µg/d siponimod group. For this group, one mouse was excluded as an outlier. Data are shown as mean±SEM. Data were analyzed for normality with Shapiro-Wilk test and for significance with Kruskal-Wallis test with post-hoc analysis using Dunn`s multiple comparisons test. Significances are depicted as * p<0.05; **** p<0.0001.


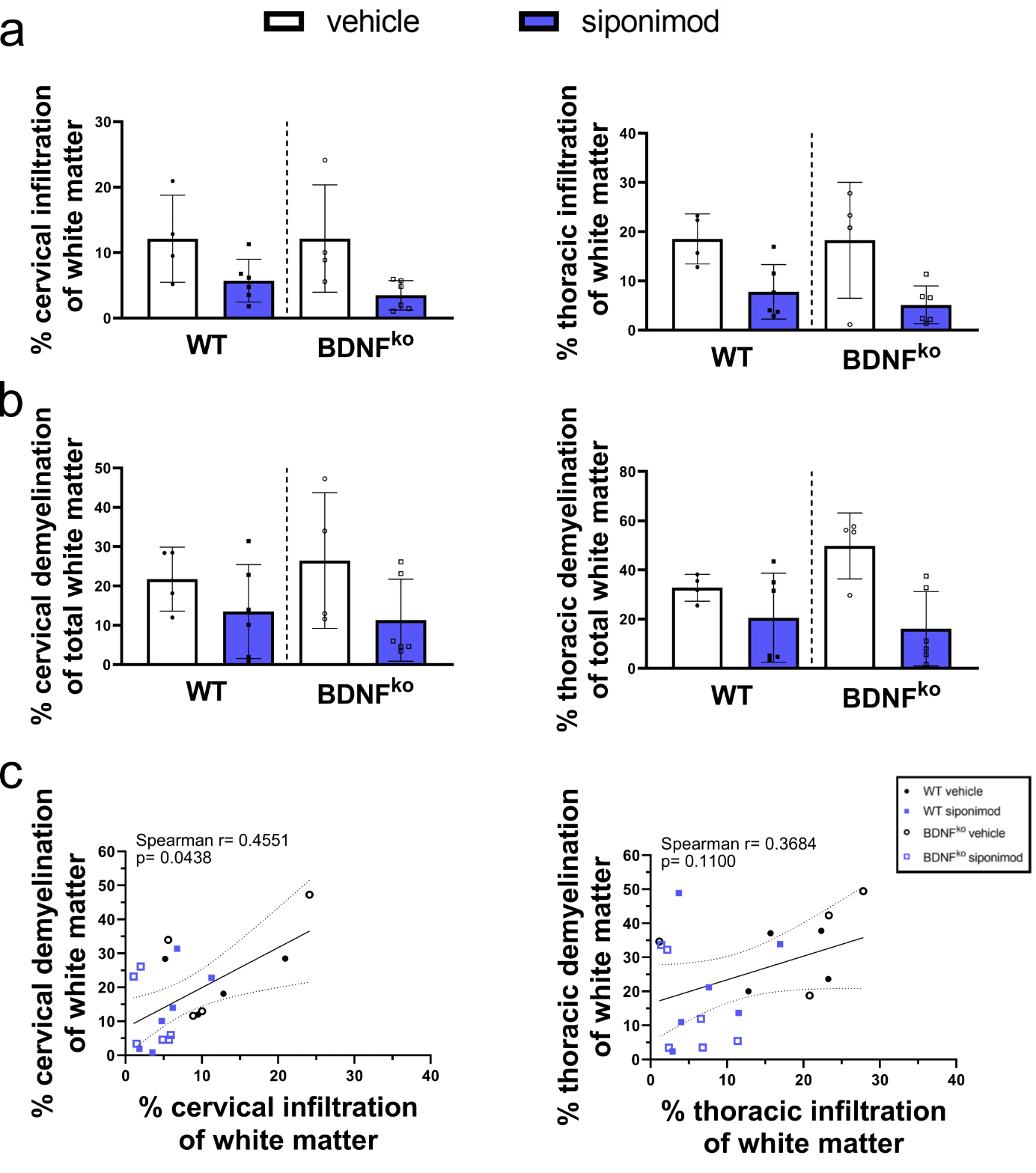


**Figure S3: Spinal cord histology of experimental autoimmune encephalomyelitis of 0.45 µg/d siponimod or vehicle-treated WT and BDNF^ko^ mice using H&E and LFB staining, related to Figure 4.** Experimental setup is presented in Figure 3a. a) Infiltration of white matter showed trends of reduction in every group after siponimod treatment. b) Demyelination of white matter seems to be reduced after siponimod treatment for WT and BDNF^ko^ mice in each compartment. For vehicle groups, the demyelinated white matter area increases towards caudal regions, being lowest in cervical area (21.7% - 26,5% (± 4% - 8,6%)) and highest in lumbar area (figure 4d, 29.6% - 36,3% (± 4,5%  - 6,6%)). c) Spearman correlation between demyelination and infiltration of white matter revealed a significant correlation for cervical spinal cord with Spearman`s r=0.4551. There was no correlation for thoraic spinal cord. Cervical and thoracic sections of spinal cords were analyzed in quadruplicates for each mouse. WT vehicle: n=4, WT siponimod: n=6, BDNF^ko^ vehicle: n=4, BDNF^ko^ siponimod: n=6. Two mice without signs of EAE (WT vehicle: n=2) and two mice with premature exitus (BDNF^ko^ vehicle: n=2) were excluded. Data are shown as mean±SEM. Infiltration and demyelination data (a, b) were analyzed for normality with Shapiro-Wilk test and tested for significance with Kruskal-Wallis test with post-hoc analysis using Dunn`s multiple comparisons test. Correlation analysis (c) was performed using Spearman correlation with 95% confidence bands. Significances are depicted as * p<0.05; ** p<0.01; *** p<0.001; **** p<0.0001.


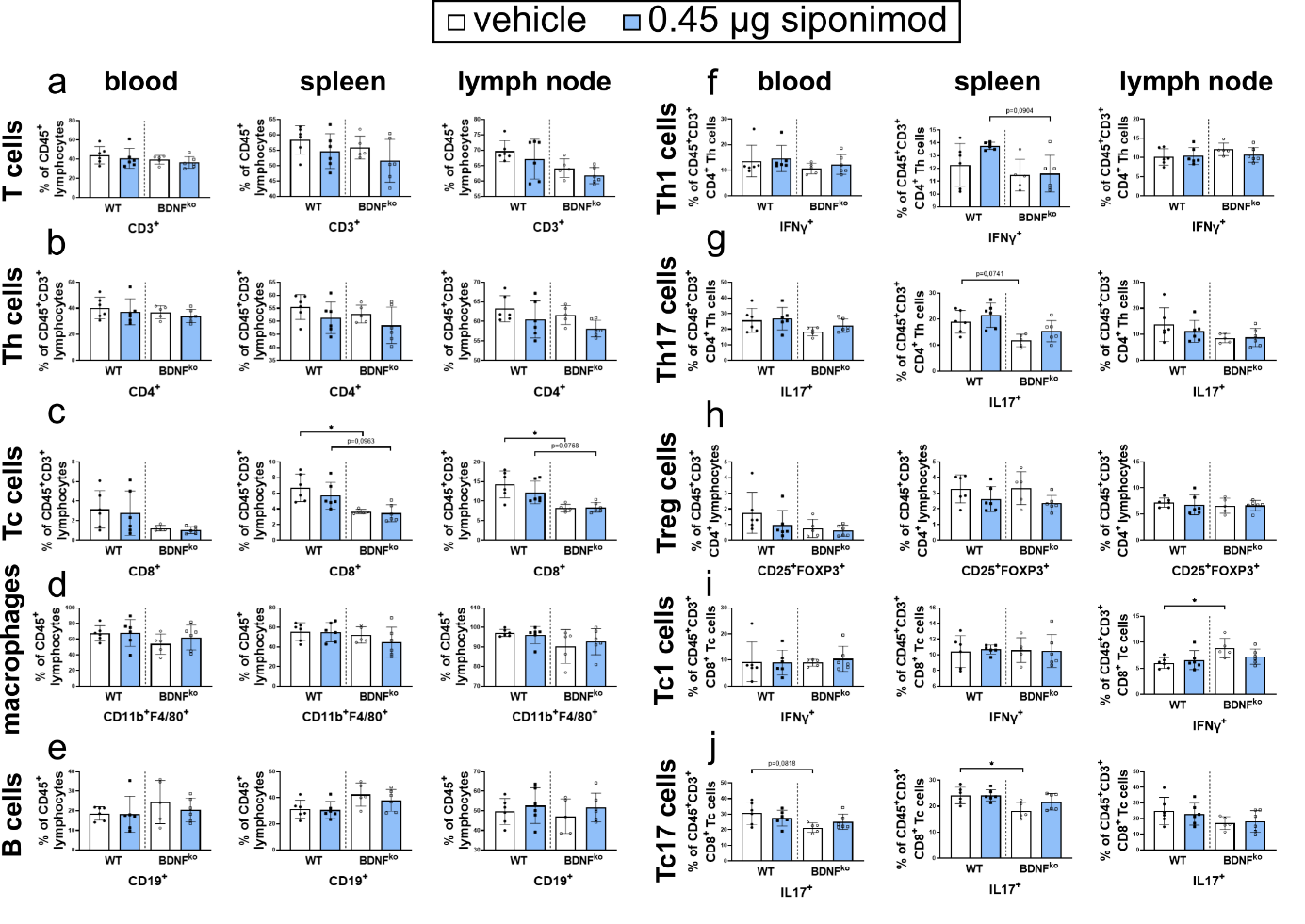


**Figure S4:** **Cells from blood, spleen and lymph nodes of 0.45 µg/d siponimod or vehicle-treated WT and BDNF^ko^ mice were analyzed via FACS.** Consistent with data shown in **Figure 2**, we did not see frequency changes due to the suboptimal dosage of 0.45 µg/d siponimod. T cell subpopulations were percentage of CD45+ lymphocytes. Experimental setup is presented in Figure 3a. a) Whereas T cells, b) Th cells and c) Tc cells showed no regulation due to siponimod treatment, there was a reduction of frequencies for Tc cells in spleen and lymph nodes in BDNF^ko^ mice. d) Macrophage populations and e) B cells were not affected. f-h) The subpopulations of Th cells consisting of Th1, Th17 and T regulatory cells did not show significant changes. i) Tc1 cell frequencies were elevated in lymph nodes due to BDNF^ko^, but not siponimod treatment. j) Tc17 frequencies were reduced in spleens of BDNF^ko^ mice. WT vehicle: n=6, WT siponimod: n=6, BDNF^ko^ vehicle: n=5, BDNF^ko^ siponimod: n=6. Data of one mice with premature exitus (BDNF^ko^ vehicle: n=1) could not be analyzed. Data are shown as mean±SEM. Data were analyzed for normality with Shapiro-Wilk test and for significance with Kruskal-Wallis test with post-hoc analysis using Dunn`s multiple comparisons test. Significances are depicted as * p<0.05.


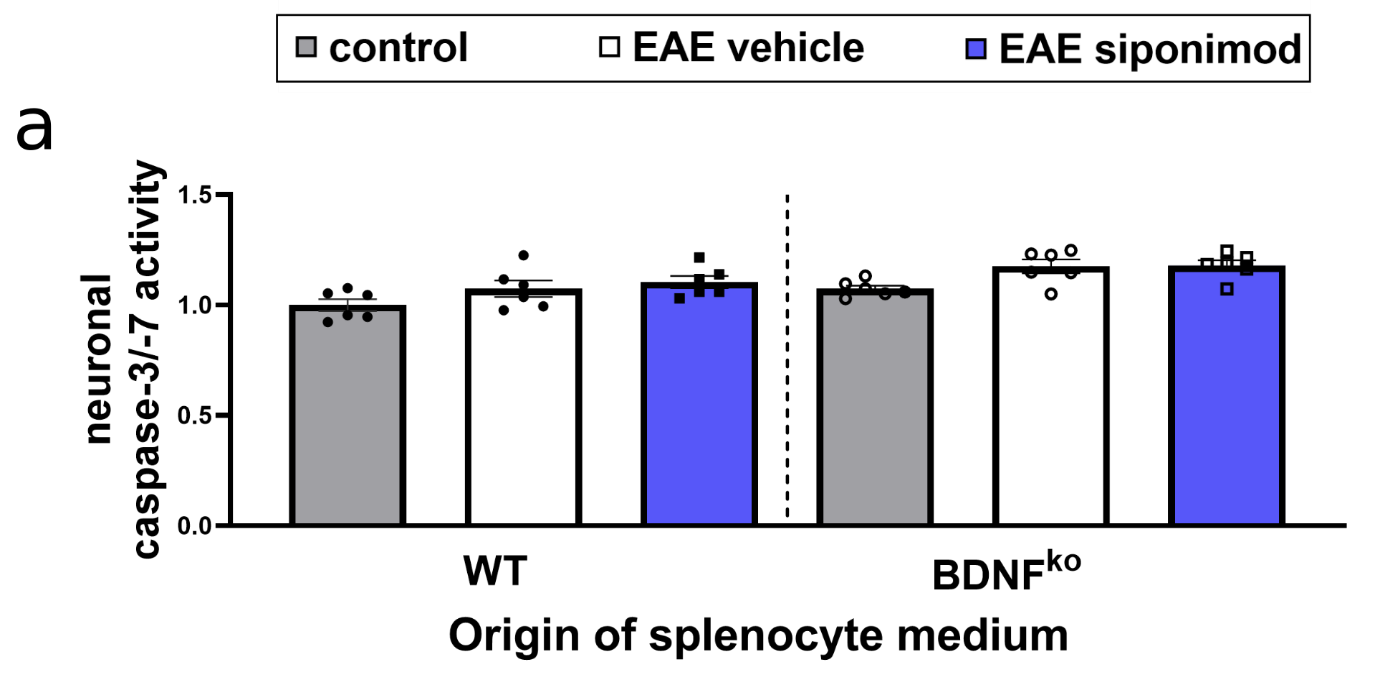


**Figure S5: 10 d after EAE-induction apoptosis markers do not show higher activity in neurons.** Supplementary data related to **Figure 5.** a) Incubation of 12.5e neurons with conditioned medium of WT splenocytes showed no neuronal caspase -3/ -7 regulation. Medium of BDNF^ko^ splenocytes seems to elicit a slight increase in caspase -3/ -7 activity for EAE, which persists despite siponimod treatment. n=3 experiments with n=2 mice are presented (n=6) performed in 4-5 replicates. Data were tested for normality with Shapiro-Wilk test and tested for significance with Kruskal-Wallis test with post-hoc analysis using Dunn`s multiple comparisons test. Significances would be depicted as * p<0.05.


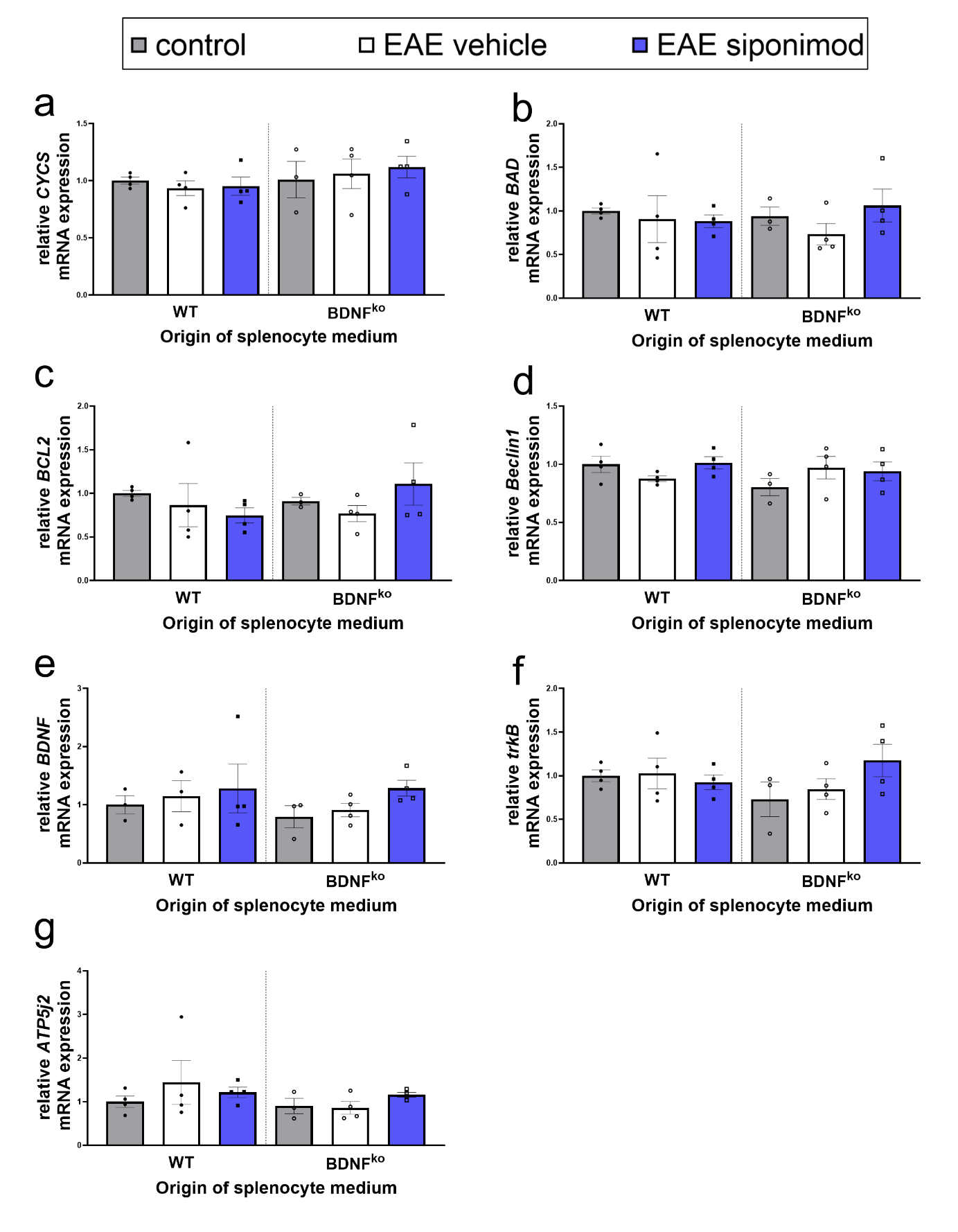


**Figure S6: Transcription of neuronal apoptosis markers was not affected following treatment with EAE conditioned media.** Experimental setup is shown in **Figure 5c**. a-c) Neuronal transcription of the apoptosis markers cytochrome c, BAD and BCL2 were not affected after incubation with splenocyte medium. d) The transcription of Beclin1, an autophagy marker, was not regulated in WT EAE mice. e) Neuronal BDNF transcription showed a trend towards an increase in BDNF-deficient medium after siponimod treatment of mice, missing significance. f) The BDNF receptor trkB was not regulated, although it showed similar trends as regulation of BDNF. g) ATP synthase markers showed no significant change. N=2 experiments with n=2 mice in each group in duplicates were performed. WT control: n=4, WT EAE vehicle: n=4, WT EAE siponimod: n=4, BDNF^ko^ EAE vehicle: n=3, BDNF^ko^ EAE vehicle: n=4, BDNF^ko^ EAE siponimod: n=4. Data of one mouse in BDNF^ko^ ctrl was contaminated. Data were normalized to their respective WT control group and shown as mean±SEM. Data were tested for normality with Shapiro-Wilk test and tested for significance with Kruskal-Wallis test with post-hoc analysis using Dunn`s multiple comparisons test. Significances would be depicted as * p<0.05.
